# A selective PanK3 activator increases coenzyme A levels in cellular and mouse models of pantothenate kinase-associated neurodegeneration

**DOI:** 10.1016/j.jbc.2026.113567

**Published:** 2026-09-17

**Authors:** Jessica Regan, Anasuya C. Pal, Shalev Gihaz, Richard E. Sutton, Choukri Ben Mamoun

**Affiliations:** 1 Section of Infectious Diseases, Department of Internal Medicine, Yale School of Medicine, New Haven, Connecticut, USA; 2 Virtus Therapeutics Corporation, Groton, Connecticut, USA; 3 Department of Microbial Pathogenesis, Yale School of Medicine, New Haven, Connecticut, USA; 4 Department of Pathology, Yale School of Medicine, New Haven, Connecticut, USA

**Keywords:** coenzyme A CoA, drug development, genetic disease, neurodegeneration, small molecule, pantothenate kinase-associated neurodegeneration

## Abstract

Pantothenate kinase-associated neurodegeneration (PKAN) is a rare progressive neurodegenerative disorder associated with iron accumulation in the brain. The disease, which causes early immobility and often death by early adulthood, is caused by loss of function mutations in the human *PANK2* gene, encoding a key enzyme in the biosynthesis of coenzyme A from pantothenic acid. To date, no disease-modifying therapy exists for PKAN, and current interventions are largely limited to symptomatic management. Herein, we report the identification of a new class of direct activators of human *PANK3*, termed VTACs, designed to compensate for the loss of *PANK2* function. Using cellular and mouse models of PKAN, we demonstrate that a lead VTAC compound, VTAC-2, increases coenzyme A levels in PanK2-deficient cells and in the globus pallidus and substantia nigra, two brain regions prominently affected in PKAN patients. Moreover, the favorable physicochemical properties and oral bioavailability of VTAC-2 make this compound an attractive candidate for clinical evaluation for the treatment of PKAN.

Pantothenate kinase-associated neurodegeneration (PKAN) is a rare, autosomal recessive disorder caused by loss-of-function mutations in the pantothenate kinase gene *PANK2* (1, 2, 3). The disease, which accounts for approximately 50% of all forms of neurodegenerative disorders associated with brain iron accumulation, typically presents in early childhood with progressive dystonia, rigidity, and loss of ambulation (4, 5, 6, 7, 8). These symptoms worsen over time to include loss of muscle control, dementia, speech impairment, and death in early adolescence (4). Neuroimaging reveals iron deposition in the globus pallidus (GP), producing the characteristic “eye-of-the-tiger” sign on MRI, which is considered pathognomonic for PKAN (9). Despite improved genetic diagnosis and symptomatic management, there are currently no approved disease-modifying therapies for PKAN, and clinical care remains largely palliative.

*PANK2* encodes pantothenate kinase 2, a key enzyme in the biosynthesis of coenzyme A (CoA) from pantothenic acid (vitamin B5) (2, 5). CoA is an essential metabolic cofactor required for a broad range of cellular processes, including fatty acid synthesis and oxidation, mitochondrial energy metabolism, and acetylation reactions (10, 11). Deficiency of PanK2 activity leads to reduced CoA availability, particularly in the brain, and is associated with secondary defects in mitochondrial function, dopamine metabolism, and iron homeostasis (12, 13, 14). These biochemical abnormalities are thought to converge to drive the selective vulnerability of neurons within the GP that characterizes PKAN pathology.

CoA is synthesized through a conserved five-step pathway, the first and rate-limiting step of which is the ATP-dependent phosphorylation of pantothenate. In humans, this reaction is catalyzed by a family of pantothenate kinase isoforms, PanK1, PanK2, and PanK3, which share ∼83% sequence homology in the catalytic domains but differ in subcellular localization, tissue distribution, and regulatory properties (15, 16, 17). PanK2 is uniquely localized to mitochondria (17, 18) and is highly expressed in neuronal populations affected in PKAN (13, 19), whereas PanK1 and PanK3 are cytosolic enzymes that remain catalytically intact in PKAN patients (15, 20). Mammalian PanK isoforms are subject to feedback inhibition by acetyl-CoA and other acyl-CoAs, thereby regulating the flux through the CoA biosynthetic pathway (16, 21, 22). These properties suggest that PKAN does not reflect a complete loss of CoA biosynthetic capacity, but rather a failure to sustain sufficient CoA production in vulnerable neuronal compartments, and that pharmacologic activation of non-mutant PanK isoforms, particularly PanK3, could compensate for PanK2 deficiency and restore CoA homeostasis. Although PanK3 is localized to the cytosol and PanK2 resides in the mitochondria, activation of PanK3 represents a promising compensatory mechanism due to the known transport of CoA into the mitochondria. CoA is synthesized in the cytosol and transported across the mitochondrial membrane through the SLC25A42 transporter (23). Consequently, enhanced production of cytosolic CoA would increase the availability for mitochondrial import, thereby restoring mitochondrial CoA levels.

On the basis of this central role for CoA deficiency in PKAN pathogenesis, multiple therapeutic strategies have been explored to restore CoA homeostasis in the nervous system. Nutritional supplementation with pantothenate or pantethine has been tested empirically (24, 25, 26, 27), but its ability to meaningfully increase neuronal CoA in the context of PanK2 deficiency remains uncertain. Iron chelation therapy, including deferiprone, can reduce brain iron accumulation but has produced variable clinical outcomes and does not directly address the underlying metabolic defect (28, 29, 30, 31). Surgical interventions such as deep brain stimulation may alleviate some motor symptoms, yet disease progression continues (32, 33, 34).

More direct approaches have focused on bypassing the defective PanK2 step by delivering downstream intermediates of the CoA pathway. A prodrug strategy designed to bypass defective PanK2 by delivering 4'-phosphopantothenate led to the development of fosmetpantotenate, which advanced to clinical trials but failed to demonstrate clinical efficacy (35, 36). Administration of 4′-phosphopantetheine has been shown to normalize CoA levels and correct iron- and dopamine-related defects in mouse models of PKAN (12, 37). This treatment, termed CoA-Z, continues to be evaluated clinically (https://nbiacure.org/coaz-clinical-trial/) (NCT04182763), underscoring the ongoing effort to overcome pathway bottlenecks while achieving adequate central nervous system (CNS) exposure.

An alternative and complementary strategy is to enhance endogenous CoA synthesis by pharmacologically activating residual pantothenate kinase activity. Small-molecule pantothenate kinase activators, known as pantazines, were developed to stabilize active PanK conformations and reduce sensitivity to feedback inhibition by CoA thioesters (38, 39). These compounds demonstrated that PanK activation can elevate cellular and brain CoA levels *in vivo*, validating PanK enzymes as druggable targets to restore CoA homeostasis. However, limitations in metabolic stability and pharmacokinetics properties have constrained the translational potential of these early pantazine compounds (38, 40).

Here, we report the identification and characterization of a new class of direct activators of human PanK3 (hPanK3), termed VTACs, designed to compensate for the loss of PanK2 function in PKAN. Using cellular models of PanK2 deficiency and mouse models with impaired PanK2 activity, we show that VTAC compounds stimulate CoA biosynthesis and increase CoA levels in PanK2 deficient cells and mouse brain tissue. A lead compound, VTAC-2, exhibits favorable drug-like properties and achieves brain exposure in mice. Importantly, VTAC-2 activation of hPanK3 was found to be independent of acetyl-CoA, unlike a previously reported hPanK3 activator, PZ-2891 and its optimized derivative BBP-671 (38, 39). Together, these findings establish direct PanK3 activation as a viable therapeutic strategy for PKAN and provide a preclinical foundation for the clinical development of VTAC-2.

## Results

### VTAC-2 selectively activates hPanK3

Our previous studies identified pyrimidone triazoles (PTZs) as a class of small-molecule modulators of eukaryotic pantothenate kinases, with minimal inhibitory activity against hPanK3 (41). These findings suggested that this scaffold could be chemically tuned to modulate isoform-specific activity. Building on this premise, we conducted a focused medicinal chemistry screen to identify compounds capable of directly activating hPanK3. Using an ATP-consumption–based assay, 113 first and second-generation PTZ analogs were systematically screened for their ability to enhance recombinant hPanK3 activity. Of the 113 PTZ analogs evaluated, 14 compounds reproducibly activated recombinant hPanK3 and were designated VTAC-1 through VTAC-14 (Table S1). These 14 compounds exhibited a broad range of potencies, with EC_50_ values spanning the nanomolar to low-micromolar range. Although multiple VTACs demonstrated measurable activation, comparative biochemical and pharmacologic profiling revealed distinct differences in potency, and predicted pharmacological and drug-like properties. Among these, VTAC-2 emerged as a lead candidate based on its activation of hPanK3, favorable physicochemical characteristics, and pharmacologic profile supportive of *in vivo* evaluation.

To define the isoform specificity and concentration–response characteristics of VTAC-2, we measured the enzymatic activity of bacterially expressed, His_6_-tagged human PanK1, PanK2, and PanK3 in the presence of increasing concentrations of VTAC-2 (0, 0.01, 0.1, and 1 μM). Dose response analyses using purified hPanK3 demonstrated strong activation with an EC_50_ of 76.5 nM. In contrast, VTAC-2 had no measurable effect on the catalytic activity of hPanK1 or hPanK2 across the same concentration range and assay conditions, confirming selective activation of the PanK3 isoform (Fig. 1, *B*, *I* and *J*).

**Figure 1:**
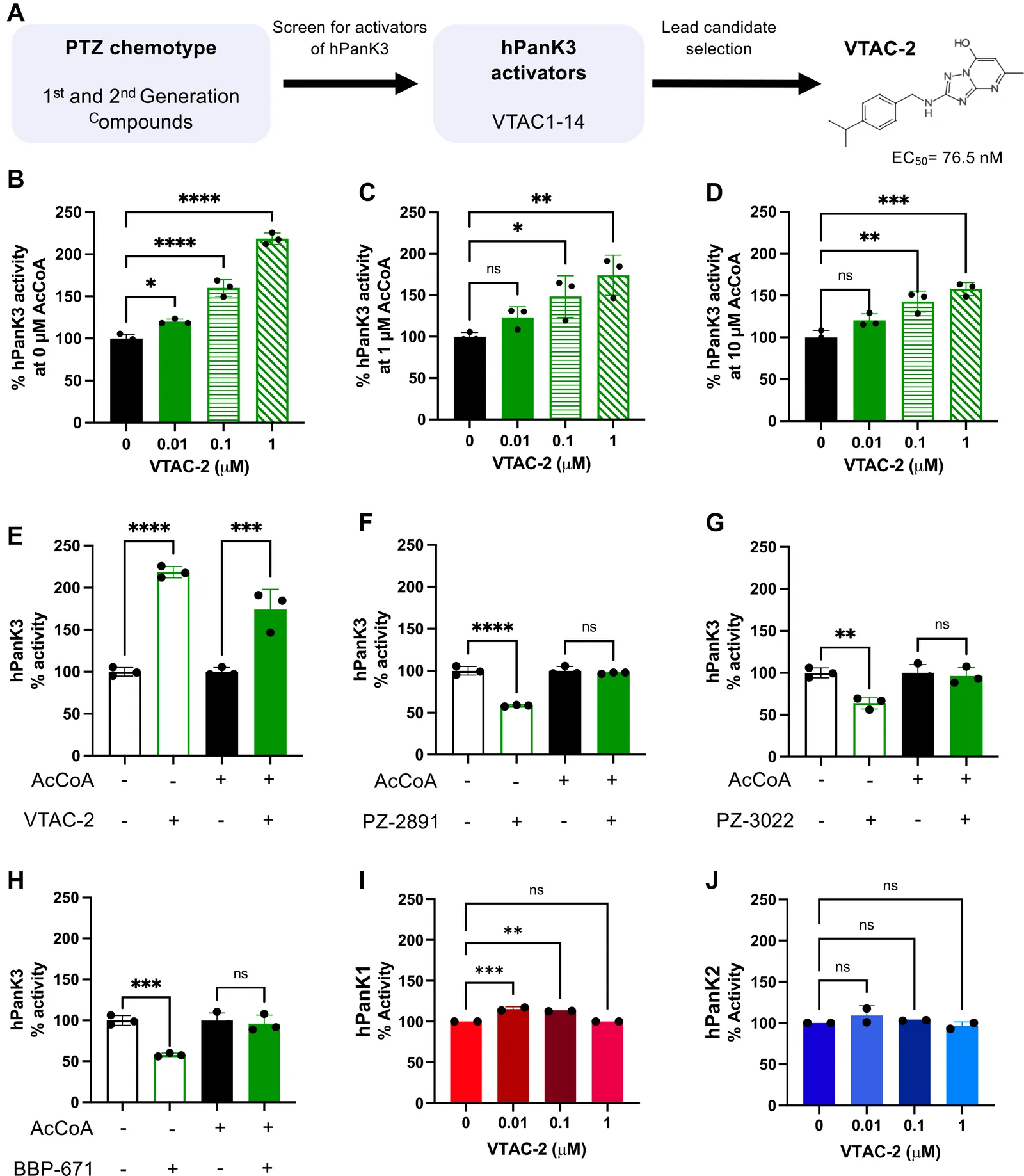
**Identification of VTACs as hPanK3 activators.***A*, summary of high-throughput screening to identify hPanK3 activators through enzymatic ATP-based assay. *B*–*D*, hPanK3 activity measured in the presence of (*B*) 0 μM, (*C*) 1 μM, or (*D*) 10 μM acetyl-CoA and VTAC-2 at 0, 0.01, 0.1 and 1 μM. E–G, and H, hPanK3 activity measured in the presence (+) or absence (-) of 1 μM acetyl CoA and 0 μM (-) or 1 μM (+) of (E) VTAC-2, (F) PZ-2891, (G) PZ3022, and (H) BBP-671. *I*, hPanK1, and (*J*) hPanK2 activity measured in the presence of DMSO or VTAC-2 at 0, 0.01, 0.1 and 1 μM in the absence of acetyl-CoA. Error bars represent mean ± SD of three replicates. Statistical significance was determined using one-way ANOVA test. ∗*p* < 0.05, ∗∗*p* < 0.01, ∗∗∗*p* < 0.001, ∗∗∗∗*p* < 0.0001. VTAC, virtus therapeutics activators of coenzyme A; hPanK3, human PanK3; CoA, coenzyme A; DMSO, dimethyl sulfoxide.

Because acetyl-CoA is both a downstream product of the CoA biosynthetic pathway and a potent feedback inhibitor of PanK enzymes, we next assessed whether VTAC-2 could activate PanK3 under varying acetyl-CoA concentrations. PanK3 activity was evaluated at 0, 1, and 10 μM acetyl-CoA in the absence or presence of increasing concentrations of VTAC-2 (Fig. 1, *B*–*D*). To directly compare the pharmacologic behavior of VTAC-2 with previously reported PanK3 orthosteric activators (39, 40, 41), we evaluated three pantazine compounds (PZ-2891, PZ-3022, and BBP-671) under identical assay conditions across increasing acetyl-CoA concentrations (Figs. 1, *E*–*H* and S1, *A*–*I*) (39, 40, 41). VTAC-2 enhanced hPanK3 activity across the full range of acetyl-CoA concentrations tested (Fig. 1, *B*–*D*). At 1 μM, VTAC-2 increased hPanK3 activity to >200% of baseline in the absence of acetyl-CoA and maintained activation at approximately 150% in the presence of 10 μM acetyl-CoA (Fig. 1, *B*–*D*). Thus, VTAC-2 elevated catalytic output substantially above the intrinsic activity of hPanK3 measured without acetyl-CoA. In contrast, pantazine compounds (PZ-2891, PZ-3022, and BBP-671) inhibited hPanK3 activity in the absence of acetyl-CoA (Figs. 1, *F*–*H* and S1, *A*–*I*) (38, 39, 40). Increasing acetyl-CoA to 10 μM mitigated this inhibition; however, under these conditions pantazines increased hPanK3 activity only to the baseline level observed for hPanK3 in the absence of both acetyl-CoA and compound, without producing net activation above that baseline (Fig. 1, *F*–*H*). Collectively, these data demonstrate that VTAC-2 drives hPanK3 activity beyond its basal catalytic state across metabolically relevant acetyl-CoA concentrations, whereas pantazines suppress hPanK3 under low acetyl-CoA conditions and fail to elevate activity above baseline even at higher acetyl-CoA. This mechanistic distinction indicates that VTAC-2 confers net catalytic enhancement independent of feedback inhibition status, a property that may be particularly advantageous in PKAN, where intracellular CoA and acetyl-CoA pools are diminished.

### VTAC-2 enhances cellular CoA biosynthesis under physiologically-relevant conditions

To evaluate the translational potential of VTAC-2, we first characterized its pharmacokinetic properties to assess systemic exposure and suitability for CNS drug development. To assess the potential impact of drug efflux on CNS penetration, VTAC-2 was evaluated using the multidrug resistance protein 1-Madin-Darby canine kidney (MDR1-MDCK) cell permeability assay. VTAC-2 exhibited an efflux ratio of 1.92, substantially lower than that of the high-efflux control digoxin (Table S3). MDR1-MDCK efflux ratios below 2.5 are generally associated with low P-glycoprotein-mediated efflux liability and are considered favorable for CNS penetration (42).

We next examined whether pharmacologic activation of hPanK3 by VTAC-2 translated into increased intracellular CoA production. Free CoA concentrations were measured in HepG2 cells, a human hepatocellular model with intact CoA biosynthesis. Plasma pantothenate concentrations are estimated to range between 1 to 2 μM under physiological conditions (43, 44, 45). Because PTZ-derived compounds can exhibit competitive interactions with pantothenate (41), it was important to determine whether VTAC-2 could stimulate CoA production at physiologically relevant substrate concentrations. HepG2 cells were cultured in pantothenate-free medium supplemented with 1 μM pantothenate and treated with increasing concentrations of VTAC-2 (0, 5, and 20 μM) for 24 h. VTAC-2 increased total intracellular CoA in a dose-dependent manner, with significant elevations observed at both 5 μM and 20 μM (Fig. 2*A*).

**Figure 2:**
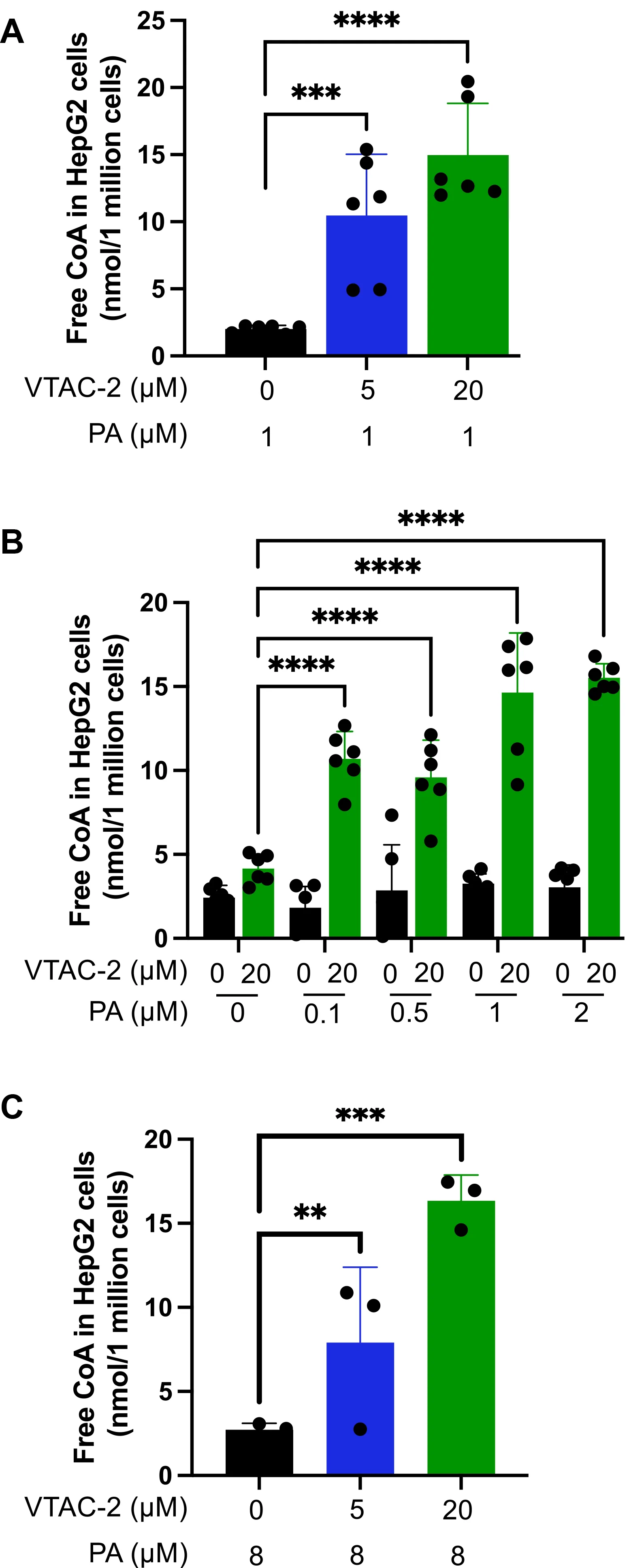
**Effect of VTAC-2on cellular CoA levels.***A*, WT HepG2 cells were grown in RPMI medium lacking pantothenate reconstituted to mimic DMEM and supplemented with 1 μM pantothenate, followed by treatment with 0 μM, 5 μM, or 20 μM of VTAC-2 for 24 h. *B*, WT HepG2 cells were grown in RPMI medium lacking pantothenate, reconstituted to mimic DMEM and supplemented with increasing concentrations of pantothenate (0, 0.1, 0.5, or 2 μM) for 3 days. Cells were then treated with 0 μM or 20 μM of VTAC-2 for 24 h. *C*, WT HepG2 cells were grown in DMEM containing 8 μM pantothenate for 3 days and treated with 0, 5 or 20 μM of VTAC-2 for 24 h. For each experiment, cell pellets were extracted and CoA levels were measured from cellular lysates. Data represent mean ± SD of three independent replicates normalized to CoA levels in nontreated cells (0 μM drug). Statistical significance was determined using one-way ANOVA test. ∗∗*p* < 0.01, ∗∗∗*p* < 0.001, ∗∗∗∗*p* < 0.0001. VTAC, virtus therapeutics activators of coenzyme A; CoA, coenzyme A; DMEM, Dulbecco's Modified Eagle Medium.

To further assess pantothenate dependence, cells were cultured in pantothenate-free medium supplemented with increasing concentrations of pantothenate (0, 100 nM, 500 nM, 1 μM, 2 μM, 10 μM, and 50 μM) in the presence (20 μM) or absence of VTAC-2 (Fig. 2B and S2). CoA production increased with rising pantothenate concentrations up to the physiological range (1–2 μM), and VTAC-2 enhanced CoA levels under these conditions (Fig. 2*B*). These data indicate that VTAC-2–mediated activation of CoA synthesis is substrate dependent, but remains effective at physiologically relevant pantothenate concentrations.

Because many PKAN patients receive pantothenate supplementation, we next evaluated VTAC-2 activity under supraphysiologic pantothenate conditions. HepG2 cells cultured in standard laboratory medium containing 8 μM pantothenate exhibited dose-dependent increases in CoA upon VTAC-2 treatment (Fig. 2*C*), reaching levels comparable to those observed at 1 to 2 μM pantothenate. Interestingly, when pantothenate concentrations were increased (10–50 μM), VTAC-2–mediated enhancement of CoA production was attenuated, and CoA levels were not significantly different from untreated controls (Fig. S2). This reduction in responsiveness at high pantothenate concentrations is consistent with feedback regulation within the CoA biosynthetic pathway under substrate-excess conditions.

### VTAC-2 stimulates CoA production in engineered and patient-derived PanK2-deficient cells

Following the observation that VTAC-2 stimulated CoA production in WT cells, we next assessed whether it could increase CoA synthesis in the context of PanK2 deficiency. To model PKAN in a controlled system, we generated CRISPR-Cas9–edited PANK2 KO human foreskin fibroblasts (HFFs), producing isogenic PANK2^+/+^ (WT) and PANK2^−/−^ (KO) cell lines (Fig. 3*A*; Table S2). Quantitative RT-PCR and immunoblot analyses confirmed marked reduction of PANK2 transcripts and complete absence of PanK2 protein in KO cells (Fig. S3, *A* and *B*).

**Figure 3:**
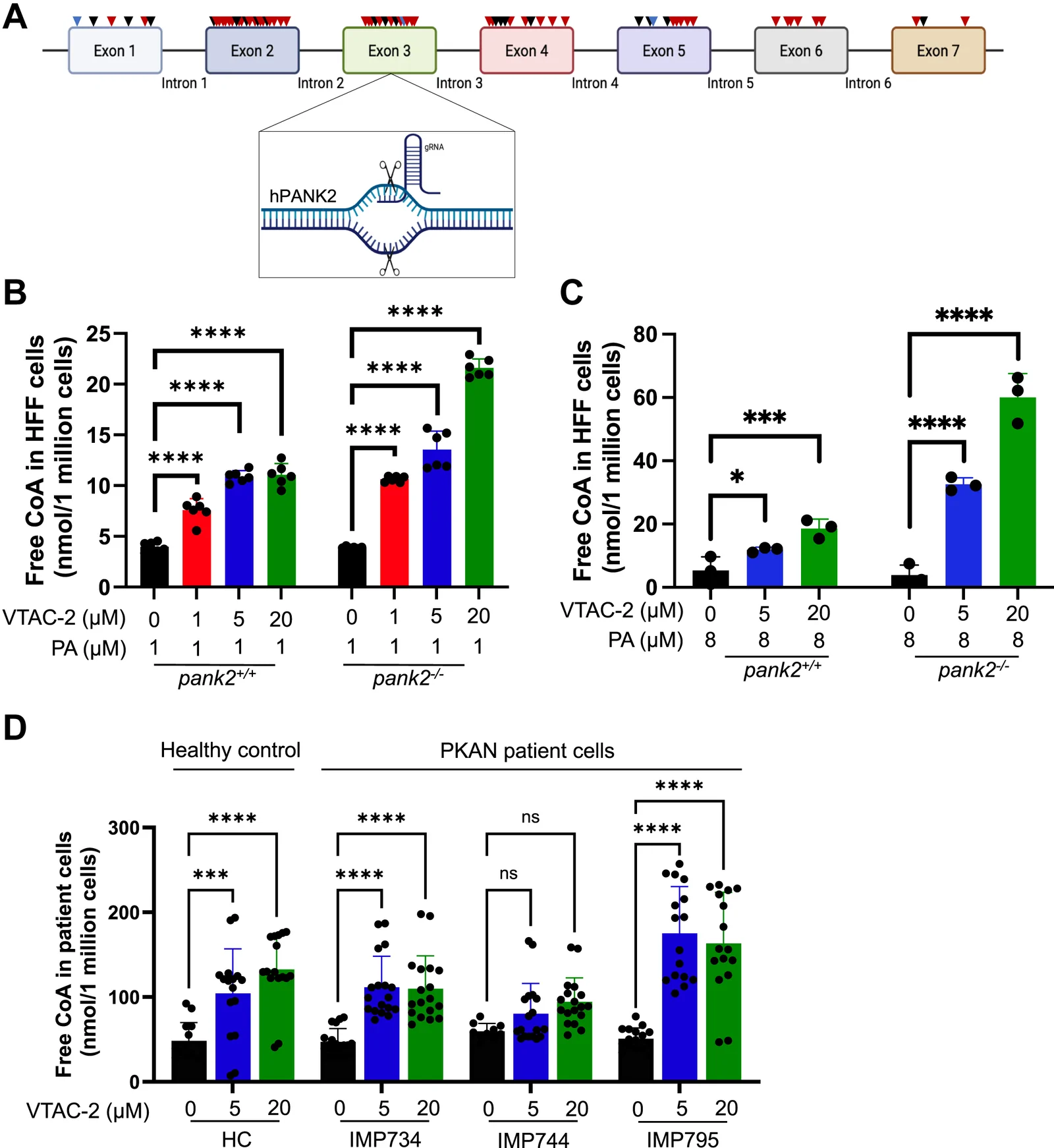
**VTAC-2 elevates CoA in PanK2-deficient cells.***A*, diagram depicting the large variety of mutations across the seven exons in *PANK2*. Exon 3 was targeted with CRISPR Cas9 to generate PanK2-deficient HFF cells (KO). *Black arrows* indicate amino acid deletions, *blue arrows* indicate frameshift mutations, and *red arrows* indicate missense mutations. Guide RNA sequences used are 5′ACTCAGTCGGATTCAATGGA3′ and 5′GATAATTACAAACGGGTCAC3′. B and C, WT HFF and PanK2-deficient HFF cells were grown in (B) RPMI medium lacking pantothenate, reconstituted to mimic DMEM and supplemented with 1 μM pantothenate, or (C) DMEM medium, containing 8 μM pantothenate for 3 days and treated with 0 μM, 1 μM, 5 μM, or 20 μM of VTAC-2 for 24 h, as indicated in the figure X-axis. Cell pellets were collected and CoA quantified from cell lysates and compared to the WT control. *D*, patient cell lines (HC and IMP) were grown in DMEM containing 8 μM pantothenate and treated with 0 μM, 5 μM, or 20 μM of VTAC-2 for 24 h. Data represent mean ± SD of three independent replicates normalized to CoA levels in nontreated cells (0 μM drug). Statistical significance was determined using one-way ANOVA test for each cell line with respect to the DMSO control. ∗*p* < 0.05, ∗∗*p* < 0.01, ∗∗∗*p* < 0.001. VTAC, virtus therapeutics activators of coenzyme A; CoA, coenzyme A; HFF, human foreskin fibroblast; DMEM, Dulbecco's Modified Eagle Medium; HC, healthy control (IMP797); IMP, immortalized patient cell lines (IMP734, IMP733, IMP768 and IMP795).

To evaluate broader effects on the CoA biosynthetic network, transcript levels of PANK1, PANK3, and COASY were quantified in WT and KO cells, along with TFRC, a marker associated with iron dyshomeostasis. PANK2-deficient HFF cells exhibited reduced transcript levels of PANK1 and PANK3, as well as altered TFRC transcript level relative to WT cells (Fig. S4, *A*–*D*), consistent with perturbations in CoA homeostasis and iron-related pathways observed in PKAN.

We then quantified intracellular CoA concentrations following VTAC-2 treatment. WT and KO HFF cells were cultured at physiological pantothenate concentrations (1 μM) and treated with increasing concentrations of VTAC-2 (0, 1, 5, and 20 μM) for 24 h. VTAC-2 increased intracellular CoA in a dose-dependent manner in both WT and KO cells (Fig. 3*B*). Importantly, CoA levels in KO cells were significantly elevated relative to untreated controls, demonstrating that activation of PanK3 by VTAC-2 compensates for loss of PanK2 function under physiologically relevant substrate conditions. We next examined whether VTAC-2 retained activity at 8 μM pantothenate. Under these conditions, VTAC-2 produced dose-dependent increases in CoA in both WT and KO HFF cells, consistent with results obtained in HepG2 cells (Fig. 3*C*).

To extend these findings to a clinically relevant context, we evaluated four immortalized fibroblast lines derived from PKAN patients alongside an age-matched healthy control (Table S2). Quantitative RT-PCR confirmed reduced PANK2 transcript abundance in PKAN cell lines (Fig. S3*C*), and immunoblotting demonstrated reduced or absent PanK2 protein expression (Fig. S3*D*). Consistent with previous reports (46), PKAN fibroblasts exhibited alterations in COASY and TFRC transcript levels (Fig. S4, *E*–*H*), consistent with changes in CoA biosynthesis- and iron-related pathways (12).

Treatment of PKAN fibroblasts with VTAC-2 (0, 5, or 20 μM) for 24 h resulted in significant, dose-dependent increases in intracellular CoA compared with untreated control (Fig. 3*D*). The increase in CoA was similar to that observed in engineered PanK2-deficient cells, demonstrating that VTAC-2 enhances CoA biosynthesis in patient-derived cells with endogenous PANK2 mutations. Collectively, these data show that pharmacologic activation of PanK3 by VTAC-2 increases intracellular CoA levels in both engineered and patient-derived PanK2-deficient cells, supporting its potential as a compensatory therapeutic strategy for PKAN.

### VTAC-2 exhibits favorable pharmacokinetic and safety properties

The translational potential of VTAC-2 was evaluated through physicochemical, safety, selectivity, and pharmacokinetic analyses. VTAC-2 demonstrated physicochemical properties broadly compatible with CNS-active small molecules, including a molecular weight of 297.4 g/mol, cLogP of 2.3, and PSA of 75.3 Å^2^ (Table S4). In three human cell lines (HeLa, HepG2, and HCT116), VTAC-2 did not reduce mitochondrial activity at concentrations up to 100 μM, indicating a favorable *in vitro* safety profile (Fig. S5).

To assess potential off-target kinase interactions, VTAC-2 was profiled against 468 kinases using the KINOMEscan scanMAX platform. At 500 nM, VTAC-2 exhibited minimal detectable binding across the kinase panel, with selectivity scores of S(35) = 0, S(10) = 0, and S(1) = 0. Percent-of-control (PoC) values exceeded 50% across all kinases tested, consistent with low binding affinity under the assay conditions. These findings support a favorable kinase-binding selectivity profile, although direct enzymatic inhibition was not evaluated.

*In vitro* ADME studies showed moderate aqueous solubility (31 μM, pH 7.4), a LogD of 2.11, and high plasma protein binding (98.4%). In human liver microsomes, VTAC-2 exhibited low intrinsic clearance (CLint 19.7 μl/min/mg) and a prolonged apparent metabolic half-life (351.5 min). Among the recombinant human CYP enzymes tested, measurable turnover of VTAC-2 was observed with CYP2C19 and CYP2C8, whereas no measurable turnover was detected with CYP1A2, CYP2B6, CYP2C9, CYP2D6, and CYP3A4 (Tables S5 and S6). Caco-2 permeability assays demonstrated asymmetric transport with an efflux ratio of ∼9.6, suggesting active efflux, although this did not preclude systemic or CNS exposure *in vivo*.

Pharmacokinetic studies in mice demonstrated terminal elimination half-lives of 3.5 h following intravenous administration (1 mg/kg) and 4.1 h following oral administration (10 mg/kg). Importantly, VTAC-2 achieved measurable brain exposure following oral administration, with mean plasma and brain concentrations remaining above the biochemical EC_50_ for hPanK3 activation for at least 2 h postdose (Fig. 4, *A* and *B*), supporting CNS exposure at pharmacologically relevant concentrations. Moreover, repeated oral dosing (10–100 mg/kg daily for 15 days) was well tolerated in male and female mice, with no significant changes in body weight, behavior, hematology, or clinical chemistry parameters. Collectively, these data support the drug-like properties, kinase selectivity, metabolic stability, CNS penetration, and tolerability of VTAC-2, justifying further *in vivo* efficacy evaluation.

**Figure 4:**
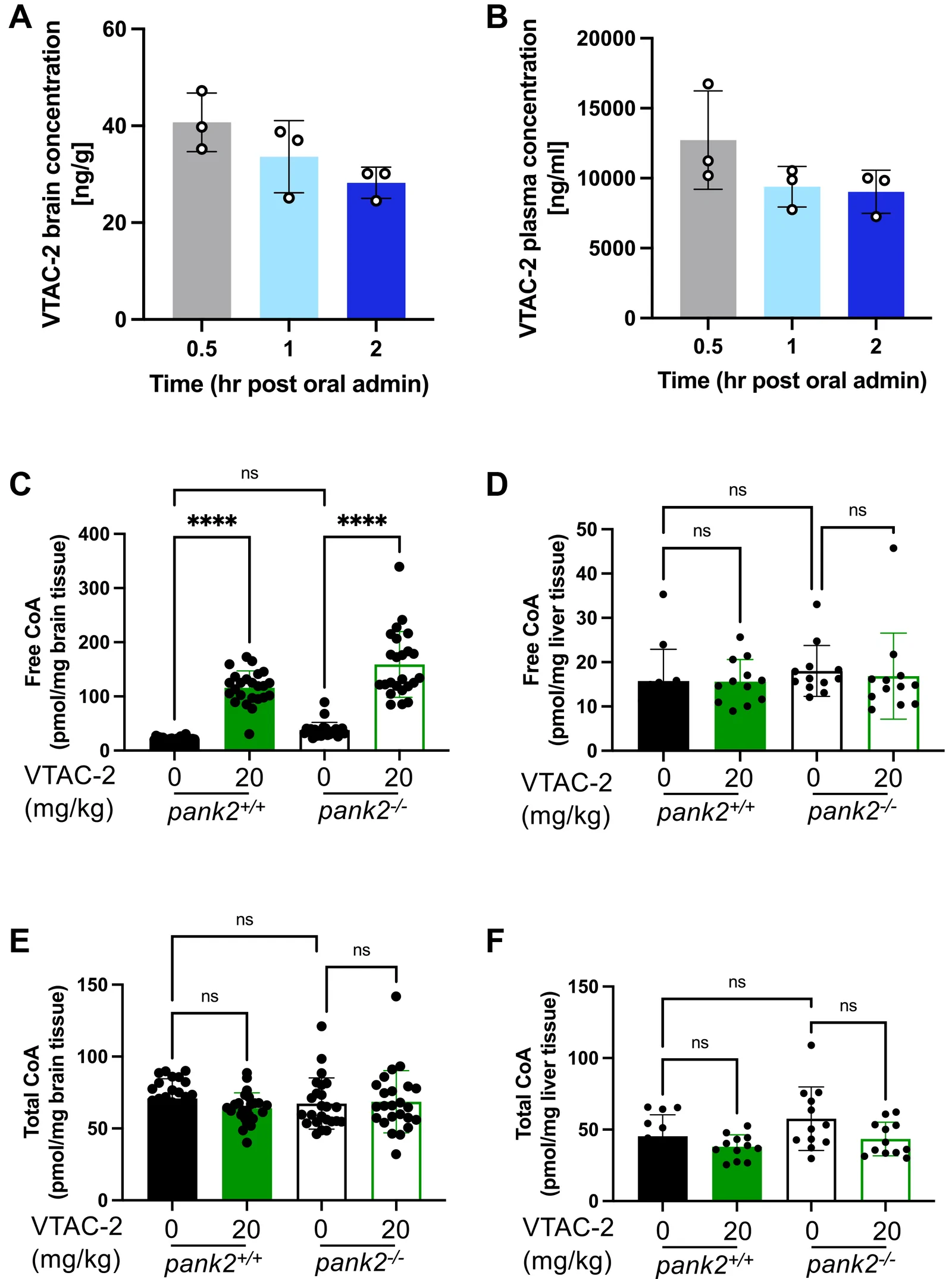
**VTAC-2 stimulates CoA production in a mouse model of PKAN.***A*, brain and (*B*) plasma concentration was measured at 30 min, 1 h, and 2 h post oral VTAC-2 dose to monitor CNS exposure. Cellular coenzyme A levels were quantified in the GP and SN regions of mouse (*C* and *E*) brain and (*D* and *F*) liver *via* the (*C* and *D*) fluorometric coenzyme A assay and (*E* and *F*) HPLC. Groups of 6 male and 6 female WT (*Pank2*^*+/+*^*)* and *Pank2*^*−/−*^ mice were treated with vehicle or VTAC-2 at 20 mg/kg *via* oral gavage for 14 days, followed by tissue harvesting. Error bars represent mean ± SD of liver samples from 12 mice and combined GP and SN samples from 12 mice. Statistical significance was determined using one-way ANOVA test for each cell line with respect to the vehicle treated control. ∗∗∗∗*p* < 0.0001. VTAC, virtus therapeutics activators of coenzyme A; CoA, coenzyme A; PKAN, pantothenate kinase associated neurodegeneration; CNS, central nervous system; GP, globus pallidus; SN, substantia nigra.

### VTAC-2 increases brain CoA in PanK2-deficient mice

To determine whether VTAC-2 enhances CoA biosynthesis *in vivo*, we evaluated its pharmacodynamic effects in whole-body KO mice lacking the PanK2 gene (12, 47). Homozygous male and female *Pank2*^*−/−*^ mice were generated from heterozygous breeders and treated alongside WT controls with vehicle or VTAC-2 (20 mg/kg, oral, once daily) for 14 days. Brain and liver tissues were harvested, with the brain tissues further dissected to enrich for GP and substantia nigra (SN) sections as previously described (12). Total RNA extracted from tissue was used in qRT-PCR to quantify transcript levels of key markers of iron and dopamine dyshomeostasis as well as the CoA biosynthetic pathway. Unlike previous reports (12, 46), we found no differences in transcript levels of key markers of iron and dopamine dyshomeostasis or genes involved in CoA biosynthesis between WT or *Pank2*^*−/−*^ mice (5, 12). This was further supported by RNA-seq performed on brain samples from WT or *Pank2*^*−/−*^ mice (Fig. S6).

Accordingly, biochemical CoA measurements were used to assess pharmacodynamic response. CoA levels were quantified in brain and liver using two independent methods: a fluorometric assay for free CoA and HPLC analysis of total CoA following hydrolysis and monobromobimane derivatization (48, 49). Baseline CoA concentrations did not differ significantly between vehicle-treated WT and *Pank2*^*−/−*^ mice (Fig. 4, *C* and *D*). Following VTAC-2 administration, the fluorometric assay demonstrated a significant increase in free CoA levels in the brain (GP and SN regions) of both WT and KO mice (approximately 5-fold relative to vehicle controls) (Fig. 4*C*). No significant changes were observed in liver tissue (Fig. 4*D*). In contrast, HPLC-based analysis of total CoA did not reveal differences among groups (Fig. 4, *E* and *F*). This discrepancy suggests that VTAC-2 may preferentially expand specific CoA pools or metabolically active fractions detectable by the enzymatic assay but not fully resolved by bulk HPLC quantification.

Together, these findings demonstrate that VTAC-2 achieves CNS exposure and increases free CoA levels, as measured by the fluorometric assay, in disease-relevant brain regions of both WT and PanK2-deficient mice. The increase in free CoA levels within these anatomically and functionally relevant brain regions provides evidence of a central pharmacodynamic response to VTAC-2 treatment and is consistent with PanK3 activation as a potential compensatory strategy for increasing CoA levels in PKAN.

## Discussion

This study establishes selective and direct activation of PanK3 as a viable pharmacological strategy to augment CoA biosynthesis in the setting of PanK2 deficiency. The catalytic domains of mammalian PanK isoforms are highly conserved, sharing ∼83% sequence homology and a common enzymatic function. Nevertheless, structural studies have identified differences in surface residues, regulatory regions, conformational dynamics, and allosteric features that can influence enzyme regulation and ligand recognition (21, 50). Despite this high degree of conservation, VTAC-2 selectively activated PanK3, with no detectable activity toward PanK1 or PanK2. This isoform selectivity suggests that VTAC-2 recognizes structural or conformational features that distinguish PanK3 from the other mammalian PanK isoforms.

The ability of VTAC-2 to selectively activate PanK3 despite the high conservation of residues within the catalytic core further suggests that its activity is unlikely to be mediated solely through interactions with the conserved catalytic site. Rather, our findings are consistent with an allosteric mechanism in which VTAC-2 directly activates catalytically competent PanK3, thereby enhancing CoA synthesis under conditions of reduced substrate availability and feedback inhibition. This interpretation is further supported by the distinct pharmacological behavior of VTAC-2 compared with previously described pantazines across varying acetyl-CoA concentrations.

VTAC-2 increased PanK3 activity even in the absence of acetyl-CoA and sustained activation at higher acetyl-CoA concentrations. This profile indicates that VTAC-2 confers net catalytic enhancement rather than simply relieving feedback inhibition, potentially enabling increased pathway flux across a broader range of metabolic conditions.

Although the concentration-dependent activation of purified recombinant hPanK3 by VTAC-2 provides strong evidence for a direct functional interaction between the compound and enzyme, the molecular basis of this interaction remains to be defined. Structural and biophysical studies are underway to characterize VTAC-2 binding to PanK3 and determine the molecular basis for its activation and isoform selectivity. Initial crystallization studies have yielded hPanK3–VTAC-2 crystals; however, these crystals did not diffract to sufficient resolution to allow structure determination. Future studies aimed at characterizing the molecular interaction between VTAC-2 and PanK3 are warranted.

Cell-based studies further demonstrate that PanK3 activation translates into measurable increases in intracellular CoA in both engineered KO models and patient-derived fibroblasts. These findings indicate that pharmacologic activation of PanK3 is sufficient to overcome the metabolic consequences of PANK2 loss at the cellular level, and further suggest that enzymatic flux through the first committed step remains a critical control point in CoA homeostasis.

An important limitation of the present study is the absence of neuronal PANK2-deficient cell models. This work was designed as a proof-of-concept study to establish the ability of VTAC-2 to directly activate PanK3 and restore intracellular CoA levels using complementary biochemical, cellular, and *in vivo* models. Although these findings establish the biochemical basis for PanK3 activation by VTAC-2, studies in neuronal PANK2-deficient models will be important to determine whether this mechanism can restore CoA homeostasis in a cellular context that more closely recapitulates PKAN. Such studies will also enable evaluation of the effects of VTAC-2 on neuronal metabolism, mitochondrial function, and disease-relevant cellular phenotypes. Additional genetic studies will be important to establish the contribution of PanK3 to the cellular response to VTAC-2. CRISPR-Cas9-mediated disruption or knockdown of PANK3, complemented by independent approaches such as siRNA, could determine whether depletion of PanK3 attenuates VTAC-2-mediated restoration of intracellular CoA. Genetic validation in cellular models would therefore provide additional evidence linking PanK3 activation to the cellular effects of the compound.

*In vivo*, VTAC-2 achieved brain exposure following oral administration and increased CoA levels in the GP and SN, regions most closely associated with PKAN pathology. The ability to elevate CoA within these anatomically and functionally relevant brain regions provides direct biochemical evidence of central target engagement and supports PanK3 activation as a compensatory strategy for restoring CoA homeostasis in PKAN. Notably, the increase in CoA was observed in both WT and Pank2-deficient mice, indicating that pharmacological activation of PanK3 can augment CoA production beyond basal homeostatic regulation *in vivo*.

The selective increase in CoA observed in brain, but not liver, is consistent with tissue-specific differences in pantothenate kinase biology. In PKAN, loss of PanK2 is most relevant to CNS CoA homeostasis, whereas hepatic CoA levels are maintained predominantly by other pantothenate kinase isoforms. As a result, additional activation of PanK3 may have limited impact in liver, where compensatory enzymatic activity is sufficient to sustain basal CoA production. The preferential pharmacodynamic response observed in the GP and SN therefore supports the concept that PanK3 activation exerts its greatest effect in tissue compartments most directly affected by disease.

Two analytical approaches were used to quantify CoA levels *in vivo*. Whereas the fluorometric assay detected a significant increase in free CoA in the GP and SN following VTAC-2 treatment, HPLC-based analysis of total CoA did not distinguish treatment groups under the conditions tested. One possible explanation is that VTAC-2 preferentially increases the free CoA pool, which would be captured by the fluorometric assay but not necessarily reflected in measurements of total CoA. This distinction is important because steady-state metabolite abundance does not necessarily reflect the activity or capacity of the underlying metabolic pathway. The total CoA pool represents the net balance among CoA biosynthesis, utilization, recycling, and degradation across multiple CoA species and may remain relatively stable despite changes in metabolic flux that alter free CoA levels. In contrast, changes in the free CoA pool may more directly reflect increased CoA biosynthesis following PanK3 activation. The detection of increased free CoA is therefore consistent with a pharmacodynamic response to VTAC-2 in these disease-relevant brain regions, even in the absence of a measurable change in total CoA. Further studies will be required to define how PanK3 activation affects the distribution of free and thioesterified CoA species in the brain and whether these changes translate into alterations in downstream CoA-dependent metabolic pathways.

A major challenge in PKAN therapeutic development is the limited availability of animal models that faithfully recapitulate the human neurological phenotype. Consistent with prior reports, the constitutive *Pank2*^*−/−*^ model used in this study did not exhibit robust biochemical or transcriptional abnormalities at baseline in the brain, despite its utility for assessing pharmacodynamic responses to CoA restoration (12). This phenotype differs from the progressive neurological dysfunction, basal ganglia degeneration, and movement abnormalities observed in patients with PKAN. Conversely, more severe mouse models with targeted neuronal depletion of PanK1 and PanK2 develop rapidly progressive phenotypes that likely reflect profound disruption of CoA homeostasis rather than the chronic neurodegenerative course observed in patients (38). Although these models provide insight into the consequences of severe disruption of CoA homeostasis, the magnitude and accelerated nature of the resulting phenotype do not accurately reflect the chronic disease course associated with human PANK2 deficiency.

The present study therefore focused on the pharmacodynamic effects of VTAC-2, including regional increases in brain free CoA, as translational endpoints consistent with its proposed mechanism of action. The increase in free CoA in the GP and SN following oral VTAC-2 administration provides evidence of pharmacodynamic activity despite the absence of a robust baseline phenotype distinguishing untreated Pank2^+/+^ and Pank2^−/−^ mice. Because increased CoA production is a direct pharmacodynamic consequence of PanK3 activation, it provides a means of evaluating target engagement that is less dependent on disease severity or the specific characteristics of an individual animal model. Interpretation of murine PKAN models must also consider potential species-specific differences in PanK2 biology. Several studies suggest that murine PanK2 differs from human PanK2 in subcellular localization, tissue distribution, and physiological function (15, 17, 51). These differences may contribute to the limited phenotype of whole-body Pank2-deficient mice compared with the severe, progressive neurological phenotype associated with PANK2 deficiency in humans. Assessments of therapeutic efficacy and disease rescue in murine models should therefore be interpreted with appropriate caution. Together, these limitations underscore both the need for improved animal models that more faithfully recapitulate PKAN and the importance of complementary biochemical and human cell-based studies for evaluating therapeutic strategies.

In addition to its pharmacodynamic activity, VTAC-2 demonstrated properties supportive of further preclinical development, including high kinase selectivity, metabolic stability, oral bioavailability, brain penetration, and tolerability in repeat-dose studies. These characteristics, together with its distinct mechanism of action, distinguish VTAC-2 from previously reported PanK activators and support its advancement as a lead compound within this chemical series. VTAC-2 was optimized for oral administration and achieved sufficient systemic exposure following oral dosing *in vivo*. Dedicated stability studies under gastric conditions have not yet been performed and should be incorporated into subsequent drug-development and formulation studies to evaluate VTAC-2 stability under physiologically relevant gastric conditions and further inform oral formulation development.

The VTAC-2 tolerability studies were designed as exploratory preclinical assessments intended to identify overt toxicity and dose-limiting effects rather than as comprehensive safety or toxicology studies. Accordingly, the relatively small cohort sizes may not detect infrequent adverse events or potential consequences of sustained CoA elevation following prolonged treatment. More comprehensive preclinical safety studies incorporating larger cohorts, repeated dosing, and longer treatment durations will therefore be required to characterize the safety profile of VTAC-2. Furthermore, although no overt cardiovascular abnormalities were observed in the present *in vivo* studies, dedicated cardiovascular safety studies, including hERG electrophysiology and other appropriate safety pharmacology assessments, will be required as the compound advances toward IND-enabling development.

Collectively, these findings establish direct pharmacological activation of PanK3 as a mechanism-based strategy to restore CoA biosynthesis in PKAN and provide evidence that such activation can achieve region-specific increases in brain free CoA following oral administration. By integrating biochemical target validation, restoration of CoA in PANK2-deficient human cells, and pharmacodynamic activity in disease-relevant brain regions *in vivo*, this study provides a foundation for the continued preclinical development of VTAC-2 and supports PanK3 activation as a therapeutic strategy for PKAN.

## Experimental procedures

### Cloning, expression, and purification of recombinant proteins

hPanK1, hPanK2, and hPanK3 enzymes carrying an N-terminal His6 tag were cloned into the pET28a expression vector and expressed in *E. coli* BL-21 (DE3). Cultures were grown in 2 ml LB kanamycin (50 μg/ml) overnight then diluted 100-fold in fresh medium and grown at 37 °C until growth reached A_600_ of ∼0.5. Protein expression was induced through the addition of 0.5 mM IPTG and grown overnight in 1 L cultures at RT. Cells were harvested through centrifugation at 8000*g* for 10 min at 4 °C, washed, and cell pellets collected. Cells were resuspended in binding buffer containing 20 mM Tris-HCl pH 7.4, 500 mM NaCl, 5% glycerol, 0.5 mM MgCl_2_, 10 mM imidazole, and protease inhibitor cocktail. Prior to sonication on ice using the Omni Sonic Ruptor 400 Ultrasonic homogenizer (15 s burst at 70% amplitude, 3 times, with 30 s cooling intervals), 1 mg/ml lysozyme and 250 U/μl benzonase DNAse was added to cells. Following sonication, cells were subjected to centrifugation at 16,000*g* for 20 min and soluble supernatant was used for recombinant protein purification by Ni^2+-^ nitrilotriacetic acid (Ni-NTA) affinity chromatography. Soluble protein was incubated with Ni-NTA in binding buffer for 1 h at 4 °C with gentle agitation. This solution was transferred to a column and washed using binding buffer to remove unbound proteins. Purified proteins were eluted from the column using elution buffer containing 500 mM imidazole. Imidazole was removed by gel filtration with a PD10 column and purified protein stocks were adjusted to 25% glycerol vol/vol, aliquoted, snap frozen in dry-ice ethanol bath, and stored in −80 °C until use.

### Pantothenate kinase activity assay and optimization

A Kinase-Glo (Promega V6711) assay for kinase activity was used to determine activity of the purified pantothenate kinases under different conditions. Enzymes were titrated using 60-min reaction time and substrate concentrations of 10 μM ATP (Teknova A1204) and 10 μM PA (Pantothenic Acid, Sigma D-pantothenic acid hemicalcium). All assays were run in PanK assay buffer (100 mM Tris-HCl pH 7.4, 10 mM MgCl_2_, 0.5 mg/ml γ-globulins, and 0.005% Tween 20). A total of 5 μl of enzyme ranging from 0.1 nM to 10 μM, or inactivated enzyme control (heat inactivated by boiling for 5 min), were added to 384 well plates (Corning 4513, 384 well, nonbinding surface, polystyrene, low volume, and round bottom). Reactions were initiated by addition of 5 μl of a premixed ATP + PA solution to achieve final assay concentration of 10 μM ATP and 10 μM PA. Reactions were allowed to proceed for 60 min, then 10 μl of Kinase-Glo reagent was added and incubated for an additional 30 min. Luminescence was detected using a Pherastar FSX (BMG LABTECH) plate reader. The VTAC-2 EC_50_ for hPanK3 activation was calculated using the online EC_50_ calculator from AAT Bioquest (https://www.aatbio.com/tools/ec50-calculator).

### High-throughput assay to identify PanK modulators

A total of 113 first-and second-generation PTZ compounds were screened for hPanK1, hPanK2, and hPanK3 activity. The optimized Kinase-Glo (Promega V6711) was performed to select for modulation of hPanK activity. The assay was conducted at final assay concentrations of 1, 10, and 100 nM compound, 0.8% dimethyl sulfoxide (DMSO), 10 μM ATP (Teknova A1204), 10 μM PA (Pantothenic Acid, Sigma D-pantothenic acid hemicalcium (21,210-5G-F), 10 nM hPanK3 in PanK assay buffer (100 mM Tris-HCl pH 7.4, 10 mM MgCl_2_, 0.5 mg/ml γ-globulins, 0.005% Tween 20) for 2.5 h at RT. After incubation, 10 μl of Kinase-Glo reagent was added and incubated for an additional 30 min and resulting luminescence was detected using a Pherastar FSX plate reader. Data were normalized to DMSO treated control (100%).

### Dose response profiling of screen active compounds

Dose response experiments on selected compounds were performed in at least triplicate to determine EC_50_ values for hPanK3. DMSO dilution series of compounds were prepared from highest concentration of 50 mM in EchoLDV 384 well polypropylene plates (Labcyte). A total of 20 nl of each DMSO dilution series were transferred to 384 well assay plates by acoustic dispense (Labcyte Echo 550) resulting in 100 μM highest assay concentration, and screens were performed as described previously with 0.5 μM hPanK3. Data were normalized to DMSO vehicle treatment (0% inhibition) and no enzyme control (100% inhibition). The percent effect of each screening compound on ATP depletion caused by PanK-mediated phosphorylation of PA was determined using ActivityBase (IDBS). Percent activation *versus* log [compound] was plotted, and EC_50_ values were determined using unconstrained four parameter logistic fitting (GraphPad Prism). Data reported are the average from at least three replicate experiments.

### Cytotoxicity assays of VTACs in human cells

Toxicity of VTACs was determined using HeLa, HCT116, HepG2 cell lines maintained in Dulbecco's Modified Eagle's medium (DMEM; Gibco) supplemented with 10% fetal bovine serum (FBS; Sigma-Aldrich) and 1% penicillin/streptomycin mix (Gibco). Cells were seeded at 20,000 cells per well in a 96-well plate and incubated at 37 °C overnight. Cells were then treated with VTACs in serial dilutions starting at 100 μM in 0.2% DMSO in growth medium at 100 μl per well and incubated at 37 °C for 48 h in a 5% CO_2_ incubator. Following incubation, 90 μl of medium was discarded from each well and replaced with 50 μl of medium supplemented with MTT assay reagent (BioVision, final concentration 0.2 mg/ml). The reaction plates were incubated at 37 °C for 4 h in the dark, followed by the addition of 50 μl 100% DMSO for solubilization of the formazan crystals. After incubation for 15 min, absorbance was recorded at 580 nM using Pherastar FSX plate reader. The percentage cell viability was calculated based on untreated cells (positive control: 100% growth) and 20% DMSO treated cells (negative control-100% inhibition).

### Efflux transporter assay

Bidirectional transport studies were performed by Jubilant using multidrug resistance protein 1-transfected MDR1-MDCK cell monolayers cultured for 5 days. Cells were maintained in DMEM supplemented with 10% FBS and antibiotics and seeded onto polycarbonate Transwell filter membranes at a density of 5 × 10^5^ cells/well. After 24 h, the medium was replaced, and cells were incubated for an additional 4 days before the transporter experiments were performed. Transport studies were conducted using Hanks' Balanced Salt Solution with 10 mM Hepes (pH 7.4) as both donor and receiver buffers. Test compounds (5 μM) were applied in duplicate in both apical-to-basolateral (A→B) and basolateral-to-apical (B→A) directions. Apparent permeability (P_app) was calculated using the following equation: P_app_ (cm/sec) = (V_r_/C_0_) (1/S) (dC/dt), where *V*_*r*_ is the receiver volume, *C*_*0*_ is the initial concentration, *S* is the surface area of the monolayer, and *dC/dt* is the drug peak area ratio in the receiver chamber with respect to time. Efflux ratio was determined as the ratio of P_app_ (B→A) to P_app_ (A→B). Efflux ratio of VTACs was compared to a high efflux control compound, digoxin.

### Construction of immortalized PanK2-deficient human cell lines

Human foreskin fibroblast cells were maintained in DMEM supplemented with 10% FBS, penicillin, and streptomycin. Transduction of primary epithelial cells was performed with third generation HIV vector encoding hTERT and hph, prepared by transient transfection of 293T cells, and pseudotyped with VSV G. Cells were selected on plates containing 200 μg/ml hygromycin and pooled immortalized epithelial cells were expanded and cryopreserved. These cells underwent another transduction with a third generation HIV-based vector encoding Cas9 and bsdr, pseudotyped with VSV G, followed by selection using 10 μg/ml Blasticidinlasticidin S. Blastidicin S and hygromycin-selected Cas9-expressing cells were expanded, and cryopreserved. These cells were then transduced with a third-generation, VSV G-pseudotyped HIV vector encoding a guide (g) RNA against PanK2 and puromycin resistance. The following oligonucleotides were used: PANK2-gRNA-1-U: 5′-ccggt**GATAATTACAAACGGGTCAC**GTTTAAGAGCTATGCTGGAAACAGCATAGCAAGTTTAAATAAGGCTAGTCCGTTATCAACTTGAAAAAGTGGCACCGAGTCGGTGCTTTTTTG-3′; PANK2-gRNA-1-L: 5′- AATTCAA AAA AGC ACC GAC TCG GTG CCA CTT TTT CAA GTT GAT AAC GGA CTA GCC TTA TTT AAA CTT GCT ATG CTG TTT CCA GCA TAG CTC TTA AAC GTG ACC CGT TTG TAA TTA TCA-3′; PANK2-gRNA-2-U: 5′-ccggt**ACTCAGTCGGATTCAATGGA**GTTTAAGAGCTATGCTGGAAACAGCATAGCAAGTTTAAATAAGGCTAGTCCGTTATCAACTTGAAAAAGTGGCACCGAGTCGGTGCTTTTTTG-3′; PANK2-gRNA-2-L: 5′- AATTCAA AAA AGC ACC GAC TCG GTG CCA CTT TTT CAA GTT GAT AAC GGA CTA GCC TTA TTT AAA CTT GCT ATG CTG TTT CCA GCA TAG CTC TTA AAC TCC ATT GAA TCC GAC TGA GTA-3′. The 2 pairs were cloned into pLKO.1Puro vector using the unique *Age I* and *Eco RI* restriction sites, just downstream of the U6 promoter. The sequence highlighted in bold corresponds to the gRNAs. Transduced cells were selected using 5 μg/ml puromycin, hygromycin, and blasticidin S, and characterized based on genotype and phenotype to obtain PanK2-deficient and WT control HFF cell lines. Single cell cloning of HFF-WT and HFF PanK2-KO cells were performed by cell sorting.

### CoA assay with VTAC-2 treated human cell lines

A CoA assay kit (Abcam) was used to quantify CoA production in human cell lines: HepG2, PKAN patient cells, and CRISPR-Cas9 edited HFF-WT and HFF-KO cell lines. Cells were seeded at 0.8 × 10^6^ cells/ml in 60 mm tissue culture treated plates in 3 ml of DMEM or RPMI medium lacking pantothenate then supplemented with increasing concentrations of pantothenate and supplemented with 10% FBS and 1% penicillin/streptomycin mix. After 3 days, cells were treated with increasing concentrations of VTACs or DMSO in DMEM or RPMI medium for 24 h. Cells were dissociated from the cell culture plate using Trypsin-EDTA (0.05%) (Gibco), counted using Countess II FL automated cell counter, and cell pellet collected after centrifugation at 2000 rpm for 5 min. Samples were then processed and the CoA assay was performed in a 96-well plate according to the manufacturer's instructions. CoA production was measured as OD570 nm, quantified based on a standard curve, and normalized to cell count. Data reported is the mean from at least three experimental replicates.

### Kinase selectivity screen

Kinase binding interactions for VTAC-2 were assessed using the ScanMAX KinomeScan platform (Eurofins DiscoverX), which evaluates compound binding to a panel of 468 unique human kinases. The majority of kinase-tagged constructs were produced by infecting *E. coli* BL21-derived hosts with T7 bacteriophage encoding the kinase of interest (multiplicity of infection = 0.4). Cultures were grown in 24-well blocks at 32 °C with shaking for 90 to 150 min, until lysis occurred. Then lysates were centrifuged at 6000*g* and filtered (0.2 μm) to remove cellular debris. A subset of kinases was expressed in HEK-293 cells and subsequently tagged with unique DNA barcodes for downstream qPCR detection.

Streptavidin-coated magnetic beads were incubated with biotinylated small-molecule ligands for 30 min at RT, blocked with excess biotin, and washed with blocking buffer (SeaBlock, 1% bovine serum albumin, 0.05% Tween-20, 1 mM DTT) to minimize nonspecific binding and remove unbound ligand. Binding reactions were assembled by combining DNA-tagged kinases, ligand-conjugated beads, and test compounds in binding buffer (20% SeaBlock, 0.17 × PBS, 0.05% Tween-20, 6 mM DTT). VTAC-2 was prepared as 100 X stocks in 100% DMSO and diluted into the assay at a final concentration of approximately 10 X AC_50_, 500 nM. All reactions were conducted in 384-well polypropylene plates in a final volume of 20 μl and incubated for 1 h at RT with shaking.

Following incubation, the beads were washed with wash buffer (1 X PBS, 0.05% Tween-20) and then incubated with elution buffer (1X PBS, 0.05% Tween-20, 0.5 μM non-biotinylated ligand) for 30 min at RT with shaking. The eluted DNA-tagged kinases were quantified *via* quantitative PCR, and compound binding was reported as selectivity score and the percent-of-control (PoC) relative to DMSO-treated samples. Details regarding the kinases tested in the KINOMEscan scanMAX platform are available at the Eurofins website: https://www.eurofinsdiscovery.com/.

### Absorption, distribution, metabolism, and excretion studies

Aqueous solubility (μM) was calculated by comparing the peak area of the principal analyte peak obtained from the buffer sample to that of the principal peak in a calibration standard (200 μM) prepared in an organic solvent (methanol/water, 60/40, v/v). Chromatographic purity (%) was defined as the ratio of the principal peak area to the total integrated peak area in the HPLC chromatogram of the calibration standard. A chromatogram of the calibration standard was recorded, accompanied by a UV/VIS absorbance spectrum with annotated maxima to confirm compound identity and purity. To evaluate the lipophilicity of test compounds, Log D values were determined as base-10 logarithm of the ratio of compound concentration in the organic phase to that in the aqueous phase. The total amount of compound was quantified using the peak area of the principal peak from a calibration standard (100 μM) prepared in a methanol/water (60:40, v/v) solution. To determine the compound concentration in the aqueous phase, three organic-aqueous partition samples of varying compositions were analyzed. The aqueous-phase concentrations were calculated as the volume-corrected and weighted sum of the principal peak areas across the three samples. An automated algorithm was employed to prioritize data from samples exhibiting well-resolved, quantifiable peaks. The amount of compound in the organic phase was derived by subtracting the aqueous-phase quantity from the total compound amount.

The metabolic stability of VTAC-2 was evaluated using human cytochrome P450 enzymes and liver microsomes. VTAC-2 was incubated with each CYP450 enzyme or liver microsome at 37 °C for 0, 15, 30, 45, or 60 min. The concentration of VTAC-2 at each time point was quantified *via* HPLC-MS/MS. Metabolic stability was expressed as the percentage of VTAC-2 remaining over time and was calculated by comparing the peak area of the compound at each time point to time zero. The half-life (t_1/2_) was determined from the slope of the initial linear range of the curve of percent compound remaining over time.

### *In vivo* pharmacokinetics of VTAC-2

*In vivo* pharmacokinetic studies of VTAC-2 were performed by Jubilant Biosys Ltd.

Groups of three animals received a single oral (10 mg/kg) or intravenous (1 mg/kg) dose of VTAC-2 followed by plasma collection at eight post-dose time points over 24 h. Plasma concentrations of VTAC-2 were quantified by LC-MS/MS, and pharmacokinetic parameters were determined by noncompartmental analysis. Terminal elimination half-life was calculated from the terminal phase of the plasma concentration-time profile using the 4- to 24- time points of intravenous administration and the 8- to 24-h time points for oral administration.

Maximum tolerated dose studies were performed by Jubilant Biosyc with groups of six C57BL/6 mice, consisting of three males and three females with vehicle or 10, 30, or 100 mg/kg of VTAC-1 and VTAC-2. Mice were treated with vehicle or VTAC for 15 days and observed daily for abnormal clinical symptoms based on body weight, appearance, and activity. On day 15, 1 h post last dose, animals were euthanized and whole blood was collected for hematology and plasma collected to assess compound exposure. Gross necropsy was performed on animals in each group to observe any abnormal changes in internal organs. Clinical symptoms were scored based on weight, body condition, posture, and movement. The maximum tolerated dose was defined as the highest dose administered that did not result in more than 15% body weight loss or cause marked changes in coat condition, posture, or locomotor activity. The primary objective of these tolerability studies was to identify overt toxicity and establish general tolerability. The described samples sizes were selected to ensure accurate assessment of toxicity by way of body weight, behavior, and survival, while avoiding the overuse of animals. Since these studies were not designed to detect or differentiate between minor differences, between groups, formal power calculations requiring larger numbers of animals was not prioritized. The results of these exploratory studies provided preliminary evidence of VTAC-2 tolerability under the conditions tested and informed the selection of dose levels for subsequent efficacy studies with larger sample sizes.

### CNS exposure study

A CNS exposure study with VTAC-2 was performed by Jubilant Biosys Ltd in 6 to 8 week old female C57BL/6 mice weighing 19 to 21 g. Animals were administered VTAC-2 by intravenous (IV; 1 mg/kg) or oral (PO; 10 mg/kg) routes. For IV dosing, mice were fed ad libitum; whereas animals receiving PO dosing were fasted for 4 h before administration. The IV formulation consisted of 5% DMSO, 5% Solutol-ethanol (1:1), and 90% normal saline at a VTAC-2 concentration of 0.2 mg/ml and was administered at 5 ml/kg. The PO formulation consisted of 0.25% Tween-80 and 0.5% methylcellulose in water at a VTAC-2 concentration of 1 mg/ml and was administered at 10 ml/kg. A terminal sampling design was used, with three mice per time point and nine mice per route of administration. Blood and whole brain were collected at 0.5, 1, and 2 h postdose. Blood was processed to plasma , and whole brains were homogenized in PBS before analysis. VTAC-2 concentrations in plasma and brain homogenates were quantified by LC-MS/MS.

### Ethics statement

All animal experiments were approved by the Institutional Animal Care and Use Committee at Yale University (protocol number 2024-20406). Animals were acclimatized for 1 week after arrival before the start of an experiment. Animals that showed signs of distress or appeared moribund were humanely euthanized using approved protocols.

### Mouse model of PKAN

Heterozygous *Pank2*^*+/-*^ mice were obtained from the Hayflick lab at OHSU and bred to generate the homozygous *pank2*^*−/−*^ whole-body PanK2 KO mice for *in vivo* efficacy studies. Initial genotyping of WT (*Pank2*^*+/+*^), *Pank2*^*+/−*^ and *Pank2*^*−/−*^ mice was conducted by extracting DNA from the toe clippings of the mice followed by PCR using the Bioline MyTaq Extract-PCR kit (cat # BIO-21127) following the instructions provided in the kit. The primers used in the PCR were: WT Forward, KO Forward, and Shared Reverse (Table S7). The PCR conditions used were: 1 cycle of 1 min at 95 °C, 38 cycles of 95 °C for 30 s, 61 °C for 30 s, 72 °C for 45 s and 1 cycle of 72 °C for 10 min. Expected band sizes were: 446 bp for the WT allele and 344 bp for the *Pank2* KO allele. Mouse colonies obtained at a later part of the study were genotyped at Transnetyx Inc. Groups of 12 mice, consisting of 6 males and 6 females, were housed under 12-h light cycles with free access to chow and water. Mice were treated with 0 or 20 mg/kg of VTAC-2 in PEG400 *via* oral gavage q.d. for 14 days and monitored for their activity. Following treatment, mouse brains and livers were removed, and the brains were trisected in regions containing the GP and SN. CoA from each mouse was quantified *via* HPLC and the CoA fluorometric assay following manufacturer's instructions.

### RNA-seq analysis of mouse brain samples

Total RNA was extracted from GP and SN enriched brain sections from *Pank2*^*+/+*^ and *Pank2*^*−/−*^ mice treated with vehicle (PEG400) or VTAC-2 (20 mg/kg) and subjected to RNA-seq at Yale Center for Genome Analysis. Subsequently, the sequence data were analyzed by using the Partek Flow software (Yale License). The count matrix was further processed using DESeq2 in R. We used standard DESeq2 protocol for data-analysis and compared the samples with pair-wise conditions. The plot was generated using SRplot program.

### HPLC analysis of monobromobimane derivatized CoA

Mass spectrometric measurements were performed with a Shimadzu Scientific Instruments 8060 Triple-Quad LC-MS system, equipped with a Nexera LC-40D xs UHPLC, consisting of a CBM-40 Lite system controller, a DGU-405 Degasser Unit, two LC-40D XS UHPLC pumps, a SIL-40C XS autosampler and a Column Oven CTO-40S. UV data were collected with a Shimadzu Nexera HPLC/UHPLC Photodiode Array Detector SPD M-40 in the range of 190 to 800 nm. Mass spectra were recorded with the triple quadrupole 8060 mass-spectrometer. The samples were held at 4 °C in the autosampler compartment. A total of 5 μl of each sample was injected into a sample loop and separated on a Shim-pack Scepter C18 to 120, 1.9 μm, 2.1 x 100 mm Column, equilibrated at 40 °C in a column oven. A binary gradient was used with a solvent system consisting of solvent A (water, HPLC grade Chromasolv, with 0.1% formic acid) and solvent B (acetonitrile, HPLC grade Chromasolv, with 0.1% formic acid). Flow was held constant at 0.4 ml/min and the composition of the eluent was changed over a total of 8 min. The ionization source was run in “ESI” mode, with the electrospray needle held at + or −4.5 kV. Nebulizer Gas was at 2 L/min, Heating Gas Flow at 10 L/min and the Interface at 300 °C. Dry Gas was at 10 L/min, the Desolvation Line at 526 °C and the heating block at 400 °C. Full mass spectra were recorded as Q3 scans in positive and negative ionization mode between 50 and 1200 m/z, as well as the MRM transitions in both polarities. Dwell time for all MRM events was 47 ms. PDA peak data were exported at wavelengths Ch1: 254 nm and Ch2: 389 nm after background subtraction with a blank solvent injection. Measurements and data postprocessing were performed with LabSolutions 5.135 Realtime Analysis and PostRun.

HPLC was also used for the ADME studies performed by Eurofins Discovery.

### Statistical analysis

Statistical analyses were performed using GraphPad Prism and Excel programs. Data are presented as mean ± SD, with the number of independent biological replicates indicated in the corresponding figure legends. Concentration-response data were analyzed by nonlinear regression using a four-parameter logistic model to determine the half-maximal effective concentration (EC50) of compounds. Statistical comparisons between two groups were performed using an unpaired, two-tailed *t* test while comparisons involving more than two groups were performed using one-way or two-way ANOVA. Statistical significance was defined as *p* < 0.05. When appropriate, exact *p* values and statistical tests are reported in the corresponding figure legends.

The primer sequences used in this study are listed in Table S7.

## Data availability

All data supporting the findings of this study are included in the article and its supporting information. Additional data and materials related to this study are available from the corresponding author upon reasonable request.

## Supporting information

This article contains supporting information.

## Conflict of interest

C. B. M. is the founder of Virtus Therapeutics, a biotechnology company developing compounds related to those described in this study. J. R. and A. P. were previously affiliated with Virtus Therapeutics but are no longer employed by or associated with the company. The remaining authors declare no conflicts of interest.

## Supporting information

Document S1, Figures S1-S6, Tables S1-S7

**Abbreviations**
CoA, coenzyme A
CNS, central nervous system
DMEM, Dulbecco's Modified Eagle Medium
DMSO, dimethyl sulfoxide
FBS, fetal bovine serum
GP, globus pallidus
HFF, human foreskin fibroblast
hPanK3, human PanK3
PoC, percent-of-control
PKAN, pantothenate kinase associated neurodegeneration
PTZ, pyrimidone triazole
SN, substantia nigra
