# Supplementary material for: A selective PanK3 activator increases coenzyme A levels in cellular and mouse models of pantothenate kinase-associated neurodegeneration
Source: J Biol Chem. 2026 Sep 17;302(11):113567. doi: 10.1016/j.jbc.2026.113567 (PMC13717179; doi:10.1016/j.jbc.2026.113567)
Supplement: Supplementary file 1 [file mmc1.pdf]

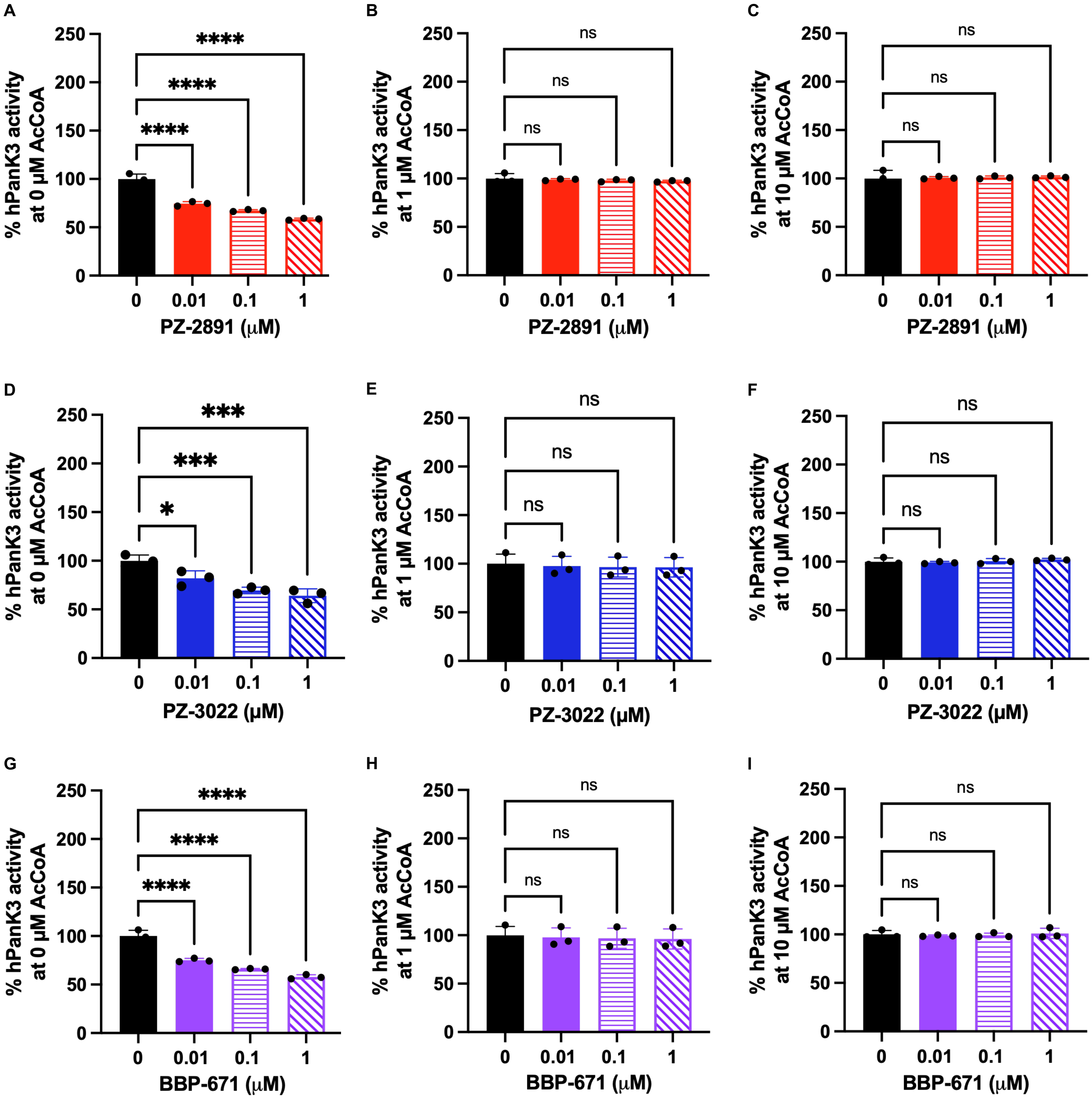

Fig. S1. Effect of AcCoA concentration on PanK3 modulation by pantazines. (A-C) hPanK3 activity measured in the presence of **(A)** 0 μM, **(B)** 1 μM, or **(C)** 10 μM acetyl-CoA and PZ-2891 at 0, 0.01, 0.1 or 1 μM. (**D-F)** hPanK3 activity measured in the presence of **(D)** 0 μM, **(E)** 1 μM, or **(F)** 10 μM acetyl-CoA and PZ-3022 at 0, 0.01, 0.1 or 1 μM. (**G-I)** hPanK3 activity measured in the presence of **(G)** 0 μM, **(H)** 1 μM, or **(I)** 10 μM acetyl-CoA and BBP-671 at 0, 0.01, 0.1 or 1 μM. Error bars represent mean +/- standard deviation of three replicates. * p<0.05, ** p<0.01, *** p<0.001, **** p<0.0001.

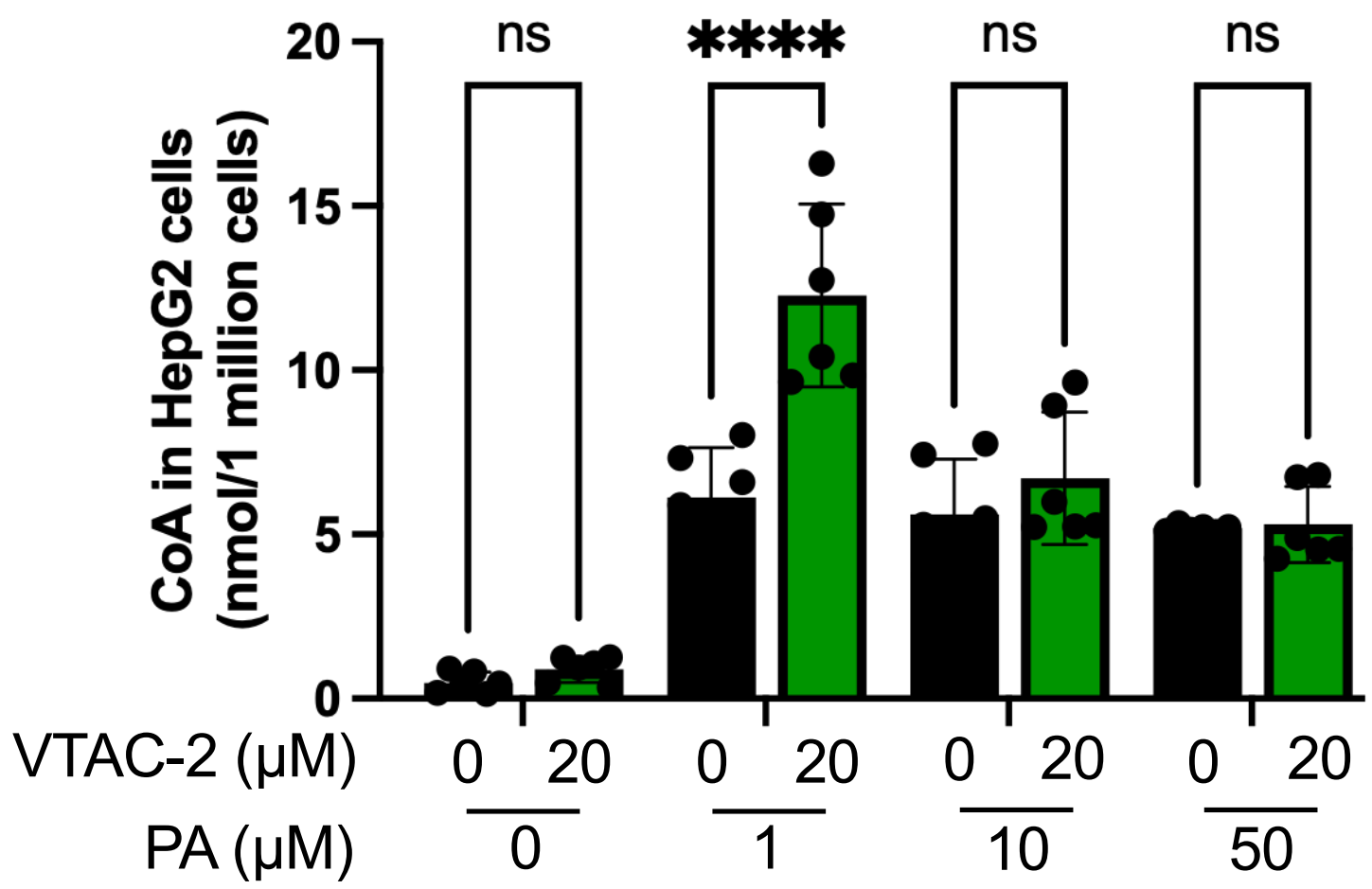

Fig. S2. Effect of VTAC-2 on CoA levels at increasing pantothenate concentrations. Wild-type HepG2 cells were grown in RPMI medium lacking pantothenate, reconstituted to mimic DMEM and supplemented with 0, 1, 10, or 50 μM pantothenate for three days and treated with 0 μM, or 20 μM of VTAC-2 for 24 hours. Cell pellets were collected and CoA quantified from cell lysates and compared to the wild-type control. Data represents mean ± SD of three independent replicates normalized to CoA levels in non-treated cells (0 μM drug). Statistical significance was determined using one-way ANOVA test. **** $p<0.0001$.

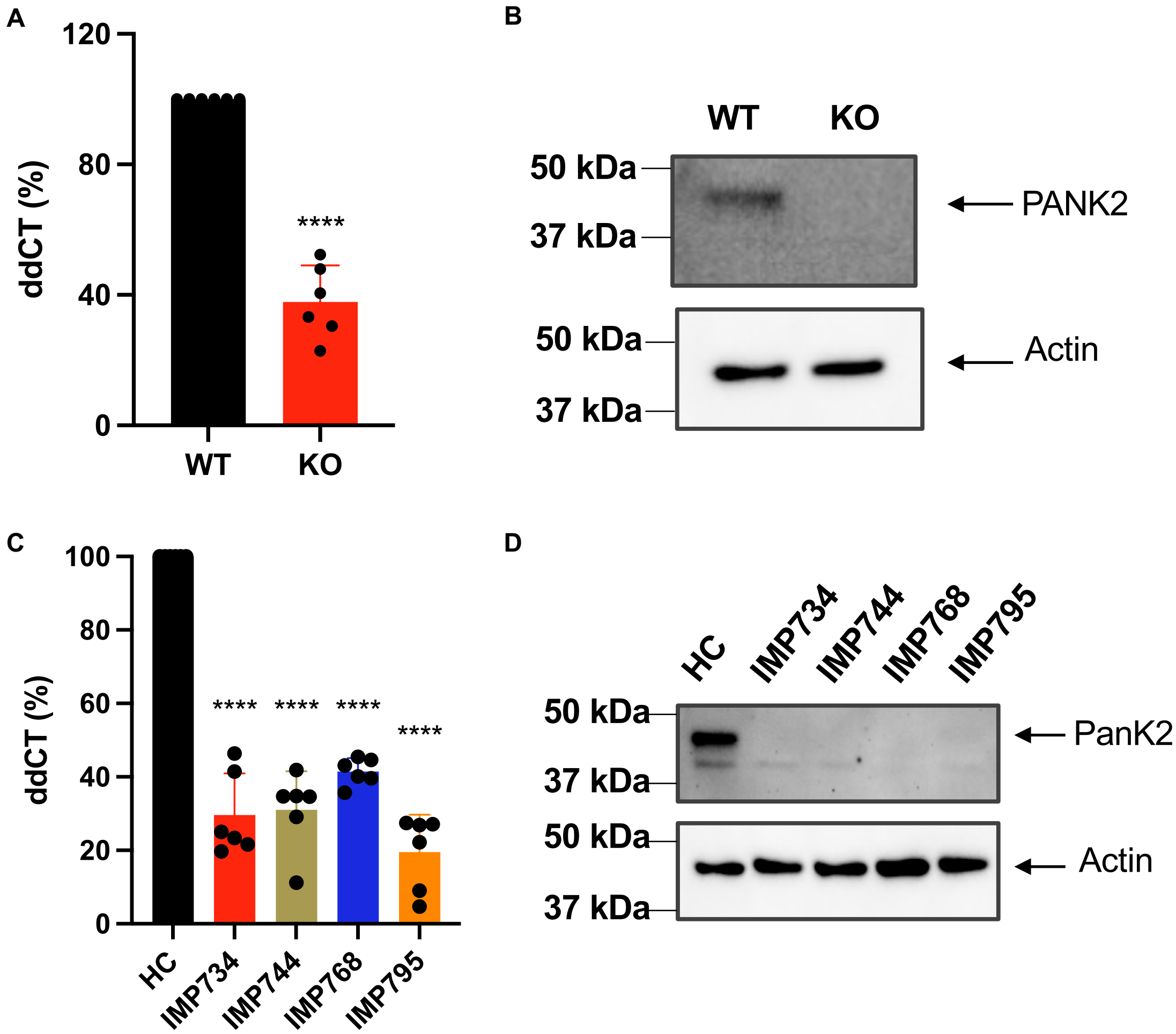

Fig. S3. *PANK2* mRNA levels and protein expression are reduced in pank2-deficient cell lines. (**A**) RNA transcript of *PANK2* was quantified via qRT-PCR and normalized to the wild-type control. Analysis was performed using the Bio-Rad CFX96 Real Time System. Error bars represent mean ± standard deviation of two independent replicates. (**B**) Western blot was performed on CRISPR-cas9-edited *pank2*$^{+/+}$ and *pank2*$^{-/-}$. (**C**) RNA transcript of *PANK2* was quantified from immortalized patient-derived PanK2-deficient fibroblasts and normalized to the wild-type fibroblast. Analysis was performed using the Bio-Rad CFX96 Real Time System. Error bars represent mean ± standard deviation of two independent replicates. (**D**) Western blot was performed on immortalized patient fibroblasts. *Note the mAb recognizes a second band whose identity is unknown. Statistical significance was determined using one-way ANOVA test. **** p<0.0001.

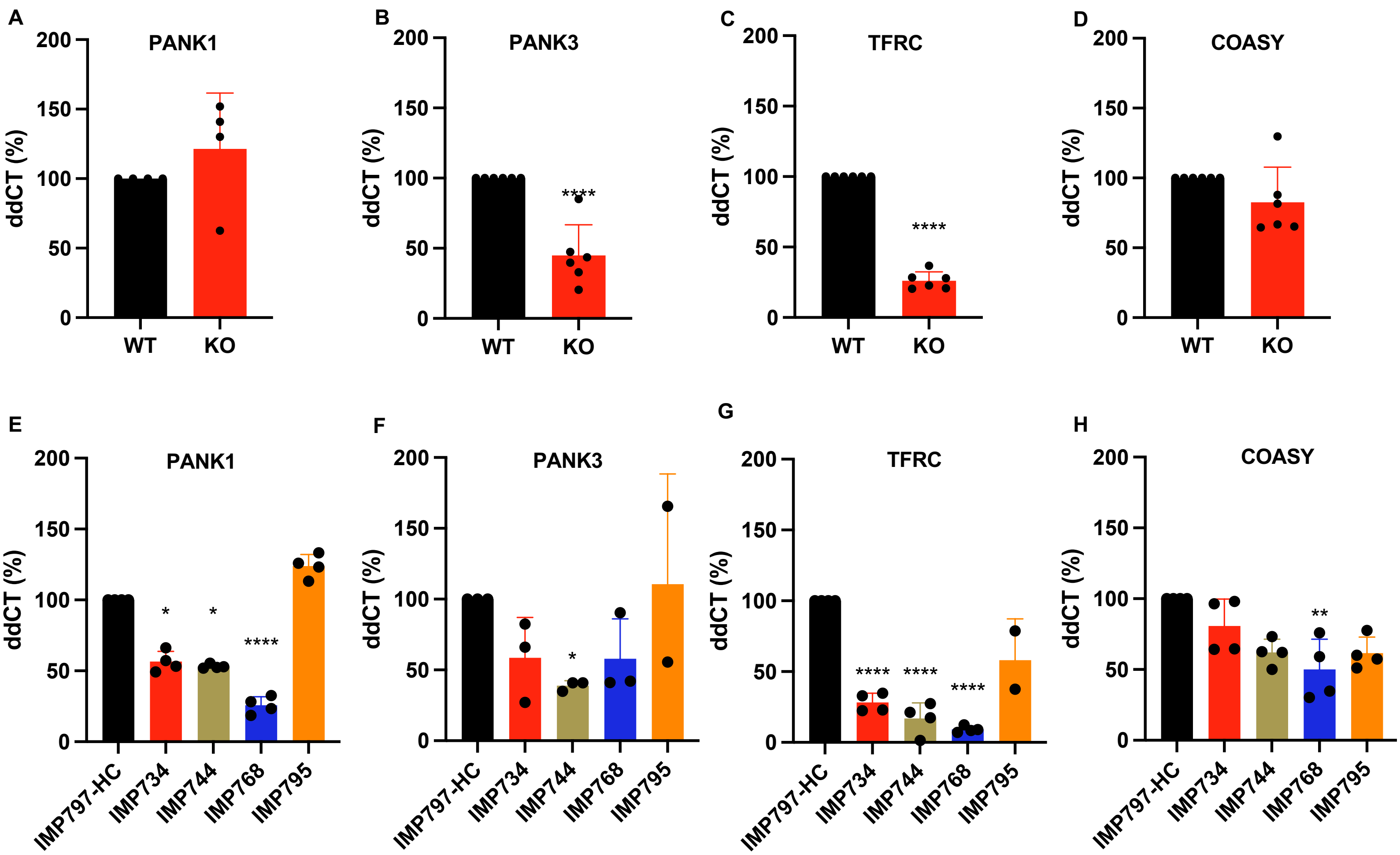

Fig. S4. Alterations of mRNA levels in *pank2*$^{-/-}$ cell lines. RNA transcript was quantified from (**A-D**) CRISPR-cas9-edited *pank2*$^{+/+}$ and *pank2*$^{-/-}$ and (**E-H**) immortalized patient-derived PanK2-deficient fibroblasts and normalized to the wild-type fibroblast. Analysis was performed using the Bio-Rad CFX96 Real Time System. Error bars represent mean ± standard deviation of two independent replicates. Statistical significance was determined using one-way ANOVA test. * $p<0.05$, **, $p<0.01$, ***, $p<0.001$.

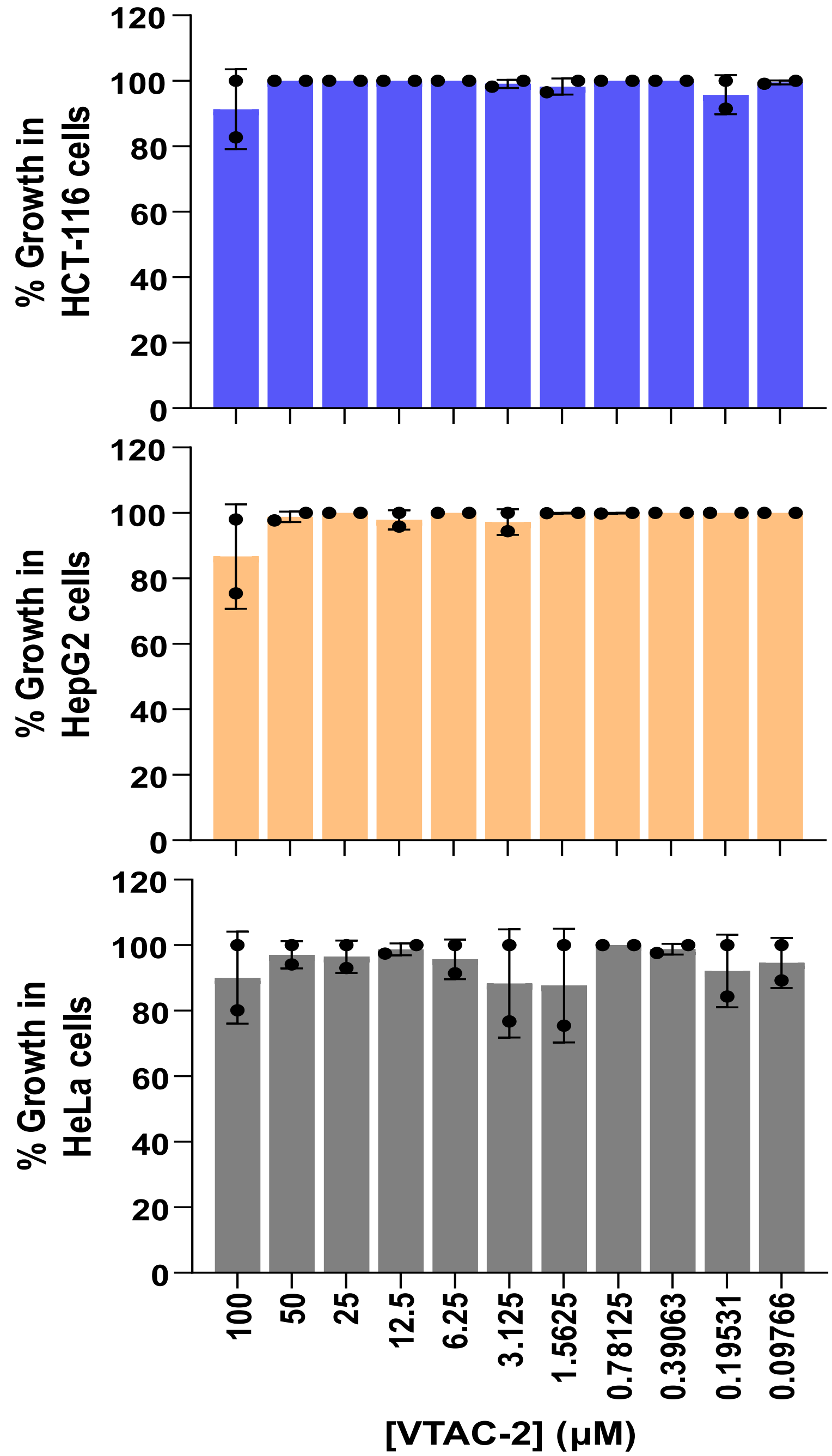

Fig. S5. Cytotoxicity of VATC-2 assessed in human cell lines. MTT assay performed on human cell lines, HCT-116, HepG2 and HeLa, treated with different concentrations of VTAC-2 for 72h, indicate no toxic effect of the drug up to 100 μM VTAC-2 concentration. Untreated cells were used as positive control (100% growth) and cells treated with 20% DMSO was used as negative control (0% growth). Experiments were performed in duplicate. Error bars represent standard deviation between replicates.

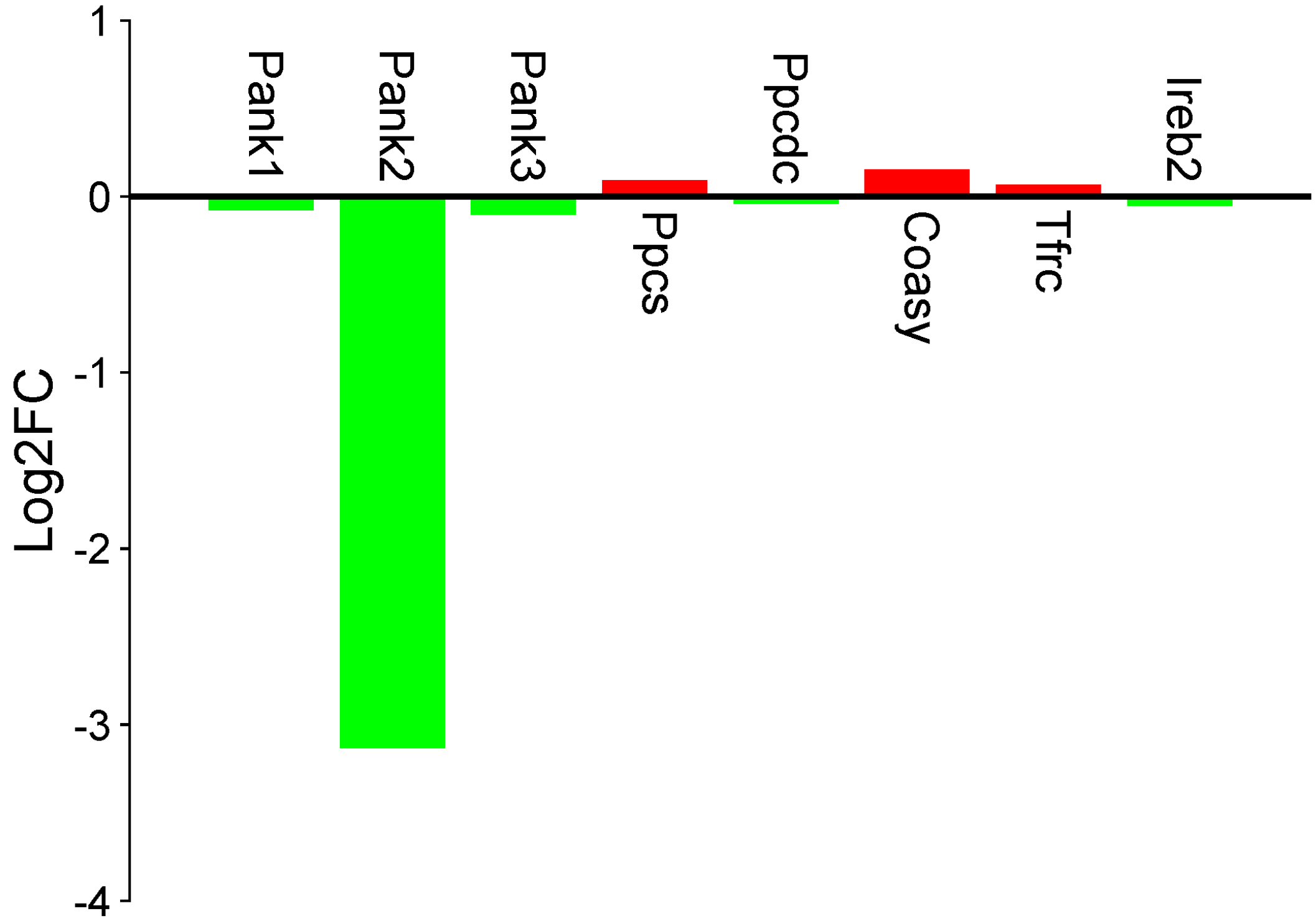

Fig. S6. Bulk RNA-seq analysis of GP enriched brain sections from WT and Pank2 KO mice. Graph shows the Log2 fold change (Log2FC) of differential gene expression of Coenzyme A pathway genes and iron regulatory genes in Pank2 KO mouse brain (3 female and 3 male) compared to that of WT mouse (3 female and 3 male). Only Pank2 transcript levels were significantly downregulated in Pank2 KO mice, while no significant differences in the transcript levels of other Coenzyme A pathway genes or iron regulatory genes were observed in Pank2 KO mouse brain compared to that of WT mouse. Positive and negative fold changes in Pank2 KO mice are indicated in red and green bars respectively. Pank1, Pantothenate Kinase 1; Pank2, Pantothenate Kinase 2; Pank3, Pantothenate kinase 3; Ppcs, Phosphopantothenoylcysteine Synthetase; Ppcdc, Phosphopantothenoylcysteine Decarboxylase; Coasy, Coenzyme A Synthase; Tfrc, Transferrin Receptor; Ireb2, Iron-responsive element-binding protein 2.

Table S1. EC_{50} values for the top 14 VTACs identified from biochemical screens.

Compound	EC_{50} (nM)
VTAC-1	44.2
VTAC-2	76.5
VTAC-3	297
VTAC-4	110
VTAC-5	223
VTAC-6	62.3
VTAC-7	578
VTAC-8	206
VTAC-9	321
VTAC-10	44.3
VTAC-11	68.1
VTAC-12	96
VTAC-13	35
VTAC-14	39.7

Table S2. Cell lines and mouse model used in this study.

**Immortalized Human Cells**	**human foreskin fibroblasts (CRISPR-Cas9 edited)**	***PANK2***$^{+/+}$
		PANK2$^{-/-}$
	Skin fibroblasts from healthy control	HC (IMP797 (*pank2*$^{+/+}$))
	Skin fibroblasts from PKAN patients	IMP734 (*PANK2*$^{-/-}$, c.1561G>A, c.1561G>A)
		IMP744 (*PANK2*$^{-/-}$, c.1561G>A, c.1561G>A)
		IMP 768 (*PANK2*$^{-/-}$, c.1319G>C, c.1319G>C)
		IMP 795 (*PANK2*$^{-/-}$, c.1561G>A, c.1537-3C>G)
**PKAN mouse model**	Whole Body *PANK2* KO	*Pank2*$^{+/+}$
		Pank2$^{-/-}$

Table S3. Bidirectional permeability of VTAC-2 in the MDR1-MDCK assay.

MDR1-MDCK permeability assay			
**VTAC #**	**P_{app} (A-B) (10^{-6} cm/s)**	**P_{app} (B-A) (10^{-6} cm/s)**	**Efflux ratio**
Digoxin	0.22	15.2	70.4
VTAC-2	20.2	38.8	1.92

Table S4. Physico-chemical properties of VTAC-2 compared to mean values, ideal range and suggested limits for the top 25 drugs used to treat neurological diseases.

Chemical Properties			
	**MW (g/mol)**	**cLogP**	**PSA (Å^2)**
Top 25 CNS drugs	293	2.8	47
Ideal Range	<450	2-4	<70
Suggested Limits	<500	2-5	<90
**VTAC-2**	297.4	2.3	75.3

Table S5. In vitro intrinsic clearance of VTAC-2 by recombinant CYP enzymes and human liver microsomes.

Metabolic system	Apparent half-life (min)	CLint
CYP1A2	NC	NC
CYP2B6	NC	NC
CYP2C19	47.2	1.5[a]
CYP2C8	48.0	0.5[a]
CYP2C9	NC	NC
Human liver microsomes	351.5	19.7[b]
CYP2D6	NC	NC
CYP3A4	NC	NC

[a] CLint expressed as µL/min/pmol CYP for recombinant CYP assays. [b] CLint expressed as µL/min/mg protein for human liver microsomes. NC, not calculable. For CYP1A2, CYP2B6, CYP2C9, CYP2D6, and CYP3A4, no consistent decrease in parent VTAC-2 was observed during the 60-min incubation. The resulting negative apparent half-life and/or intrinsic clearance estimates were therefore considered nonphysiological and are reported as NC rather than as negative values.

Table S6. VTAC-2 stability after 1 hour in CYP enzymes and liver microsomes.

CYP enzyme	Incubation time (minutes)	% compound remaining (mean)
CYP1A2	0	100
	15	110.44
	30	113.24
	45	110.82
	60	111.17
CYP2B6	0	100
	15	91.26
	30	101.04
	45	96.71
	60	101.68
CYP2C19	0	100
	15	92.42
	30	68.31
	45	54.37
	60	43.33
CYP2C8	0	100
	15	91.69
	30	69.19
	45	54.75
	60	43.75
CYP2C9	0	100
	15	152.53
	30	148.67
	45	139.96
	60	130.33
CYP2D6	0	100
	15	111.79
	30	116.50
	45	111.29
	60	114.05
CYP3A4	0	100
	15	89.25
	30	107.07
	45	102.27
	60	109.30
Liver microsomes	0	100
	15	88.27
	30	90.60
	45	88.55
	60	86.39

Table S7. Primers used in this study.

Primer	Sequence (5'→3')
PANK2F1	ATGAGGAGGCTCGGGCCC
PANK2F2	TCACAATAGGTGATCTTCAGCTTTGC
PANK2F3	CCCTGCTGATTCTGAAAAGTGTC
PANK2R3	TCCGTATCCTCTGCTTCTGGTGAA
PANK2R2	GAAGAAGCTCTTGAAATGGCATCT
PANK2R1	GAGCTGTTGAAGATCCCGTGA
qPCR PrimerF	CGTGGAGATAGCACCAAAGTGG
qPCR PrimerR	GGCTGTCAGTAAAGAGGACCTG
gRNA1	GATAATTACAAACGGGTCAC
gRNA2	ACTCAGTCGGATTCAATGGA
WT forward	TCATTCCTTTCCCCTGTGTGTA
KO forward	CCACTCCCACTGTCCTTTCCTA
Shared reverse	CCCAACTGCCTAAGCACCTACT
